# IFT20 Mediates the Transport of Cell Migration Regulators From the Trans-Golgi Network to the Plasma Membrane in Breast Cancer Cells

**DOI:** 10.3389/fcell.2021.632198

**Published:** 2021-02-26

**Authors:** Huihui Yang, Fan Zhang, Huan Long, Yiwen Lin, Jiahui Liao, Haibin Xia, Kaiyao Huang

**Affiliations:** ^1^Key Laboratory of Algal Biology, Institute of Hydrobiology, Chinese Academy of Sciences, Wuhan, China; ^2^University of Chinese Academy of Sciences, Beijing, China; ^3^The State Key Laboratory Breeding Base of Basic Science of Stomatology & Key Laboratory of Oral Biomedicine Ministry of Education (Hubei-MOST & KLOBM), Wuhan University, Wuhan, China

**Keywords:** IFT20, breast cancer cell, migration, vesicular transport, tumor suppressor

## Abstract

IFT20 is a subunit of the intraflagellar transport (IFT) system essential for the formation and function of cilia. Besides predominant research in the cilia field, some IFT subunits perform extraciliary roles in non-ciliated cancer cells. However, the specific roles of IFT subunits in tumorigenesis remain unknown. Here, we found that knockout of IFT20 in mouse breast cancer cells lacking primary cilia promoted epithelial mesenchymal transitions (EMTs), active lamellipodia formation, and cell migration. IFT20 localized at the trans-Golgi and trans-Golgi network (TGN), and displayed vesicular co-distributions with Rab8a, the marker of TGN-to-plasma membrane vesicular trafficking. Proximity-dependent biotin identification (BioID) and colocalization analyzes showed that Numb and Ctnnal1, whose depletion promoted cell migration, co-localized with IFT20 at the trans-Golgi/TGN or intracellular transport vesicles. Furthermore, Strep-Tactin pulldown assays revealed an interaction between IFT20 and Ctnnal1 or Numb. Loss of IFT20 lowered the expression of actin-associated Tagln2, whose knockdown promoted cell migration. Thus, the extraciliary function of ITF20 in breast cancer cell was associated with the negative regulation of migration.

## Introduction

Intraflagellar transport (IFT) is an active bi-directional transport system in cilia that comprises of motor proteins (kinesin 2 and cytoplasmic dynein 2) and IFT complexes, which transport the ciliary assembly blocks and signaling molecules between cilia and cell bodies (Kozminski et al., 1993; Rosenbaum and Witman, 2002; Nakayama and Katoh, 2020). The IFT complexes serve as adaptors or bridges between the ciliary cargoes and motor proteins, and are composed of at least 22 subunits (Lechtreck, 2015). Besides the known transport roles in cilia, some IFT subunits have been reported to possess extraciliary/cilia-independent functions in non-ciliated cells. For example, IFT20, IFT57, and IFT88 are required for polarized recycling of T cell receptors (TCRs) to immune synapses (Finetti et al., 2009). IFT20 controls lysosome maturation by regulating the retrograde transport of cation-independent mannose-6-phosphate receptors (CI-M6PR) (Finetti et al., 2020); additionally, it affects osteosarcoma cell migration by regulating the dynamics of Golgi-associated microtubules (Nishita et al., 2017). Previous work has also reported that depleting IFT88 perturbs cell migration by reducing the number of microtubules at the leading edge, which is independent of cilia (Boehlke et al., 2015). These extraciliary functions of IFT subunits have greatly deepened our understanding of IFT proteins. However, whether these extraciliary functions of IFT proteins are derived or different from their cilia-associated transport roles have not been fully elucidated especially in non-ciliated cancer cells.

Recent studies have indicated that IFT20, a subunit of the IFT complex and crucial for ciliogenesis (Follit et al., 2006), may have additional extraciliary functions. Different from other IFT components localized at the basal body and cilium, IFT20 is also distributed at the Golgi in ciliated cells, corresponding to its unique role in transporting the ciliary membrane proteins polycystin-2 or opsins from the Golgi to the cilia (Follit et al., 2006; Jonassen et al., 2008; Keady et al., 2011). In addition to its cilia-associated roles, IFT20 also performs several extraciliary functions, such as transporting TCRs and linkers for activation of T cell to immune synapses (Finetti et al., 2009; Vivar et al., 2016), and interacting with dynein in the retrograde vesicular traffic from the late endosome to the TGN (Finetti et al., 2020). IFT20 also participates in the vesicular transport targeted to post-synaptic dendritic terminals in neurons (Sedmak and Wolfrum, 2010) and modulates β1-integrin recycling to focal adhesions in epidermal cells (Su et al., 2020). These studies imply that cell-type-specific extraciliary functions of IFT20 exist in different cell lines.

During breast cancer progression, the occurrence of primary cilia decreases with the increasing degree of transformation (Yuan et al., 2010), which suggests that breast cancer cells without cilia might be an appropriate model for investigating the extraciliary functions of IFT20. Here, we demonstrate that knockout of IFT20 promoted non-ciliated breast cancer cell migration. Using proximity-dependent biotin identification (BioID) and Strep-Tactin pulldown assays, IFT20 was found to participate in the vesicular transport of Numb and Ctnnal1 from the trans-Golgi/TGN to the plasma membrane; moreover, both IFT20 interactors inhibited breast cancer cell migration. Interestingly, knockout of IFT20 lowered the expression of Tagln2, which took part in the dynamic regulation of F-actin. Taken together, our study has unveiled a repressor role of IFT20 in breast cancer cell migration through the cilia-independent vesicular trafficking pathway.

## Materials and Methods

### Cells, Plasmids, and Transfections

Human breast cancer cell lines (MCF-7 and MDA-MB-231), the immortalized human breast epithelial cell line HBL-100, mouse breast cancer cell lines 4T1 and IFT20 knockout 4T1 cell lines, HEK293T, mouse embryo fibroblast (MEF), and HeLa cells were cultured in Dulbecco's modified Eagle's medium (DMEM) supplemented with 10% (V/V) fetal bovine serum (FBS), 100 U/mL penicillin, and 100 μg/mL streptomycin in a humidified 37°C atmosphere containing 5% CO_2_.

The px330-mCherry plasmid (98750; Addgene) was a gift of Dr. Guoliang Xu (Institute of Biochemistry and Cell Biology, Shanghai). The Golgi marker plasmid pGT-mCherry (55052; Addgene) and F-actin marker plasmid pEGFP-C1 Lifeact-EGFP (58470; Addgene) have been previously described (Efimov et al., 2007; Riedl et al., 2008). IFT20 was amplified via polymerase chain reaction (PCR) by using the cDNA from 4T1 cells. The PCR products were digested and inserted into pHAGE-6× Tag (a gift of Dr. Yongan Zhang; Institute of Hydrobiology, Wuhan), pEGFP-N1, or pcDNA3.1-BirA*-Myc.

pGT-EGFP was created by digesting pGT-mCherry and pEGFP-N1 with *Nhe*I and *BamH*I. The pGT-EGFP-mCherry construct was created by *Nhe*I and *Kpn*I digestion of the products of PCR-amplified GT-EGFP and pmCherry-N1 with 12 amino acid between EGFP and mCherry. Rab8a was amplified using PCR from the cDNA of 4T1 cells and then digested and inserted into pmCherry-N1. pCS2(+)-Rab5a-mCherry was a gift of Dr. Yonghua Sun (Institute of Hydrobiology, Wuhan). Rab10, Rab11a, Rab11b, Rab7, Rab9, and Rab31 were amplified via PCR using the cDNA from 4T1 cells, and products were then digested and inserted into pEGFP-C1. Numb, Ctnnal1, Wwox, and Talgn2 were amplified by PCR from the cDNA of 4T1 cells and then digested and inserted into pmCherry-N1 or pEGFP-N1. pLKO.1-TRC, psPAX2, and pMD2.G were purchased from Addgene. The relevant primers used for CDS (Coding DNA Sequence) amplification are listed in Supplementary Table 1.

To generate the pcDNA3.1(-)-IFT20-Strep construct, DNA encoding a Strep-Tag II (5′-ggatccTCTGCTTGGAGCCACCCACAGTTCGAGAAAGGGGGCGGCTCCGGAGGAGGTTCCGGGGGCAGCGCCTGGAGCCATCCTCAGTTCGAGAAGTAGaagctt-3′) that also contained *BamH*I and *Hind*III restriction sites was synthesized by the Shanghai ShengGong Company and then digested with *BamH*I and *Hind*III to insert the tag at the C-terminal in the pcDNA3.1(-)-IFT20 construct.

The transient transfections of expression plasmids were carried out by using Lipofectamine 2000 reagents (Invitrogen). For lentivirus production, HEK293T cells were transfected with pHAGE or pLKO.1 and the packing vectors psPAX2 and pMD2.G. Virus-containing medium was collected 60 h after transfection and filtered with a 0.45-μm filter. Viral supernatant mixed with 8 μg/mL polybrene was used to infect the target cells over a period of 12 h, and the viral supernatant was then replaced with fresh medium. The stable cell lines were selected through treatment with 2 μg/mL (4T1 cells) or 4 μg/mL (IFT20 knockout cells, B13) puromycin for 72 h after infection. For cell lines stably expressing IFT20-Flag (B13+IFT20-Flag), following puromycin selection for 72 h, a single clone was further isolated by placing one cell per well using a fluorescent-assisted cell sorting (FACS) system and screening in the presence of 4 μg/mL puromycin. The efficiency in different cells was determined via Western blot (WB) or quantitative PCR (qPCR) method. The relevant primers used for knockdown or qPCR are listed in Supplementary Table 1.

### Antibodies and Staining Reagents

Primary antibodies used included anti-tubulin (Abcam; 1:8,000 for WB), anti-IFT20 [gift of G. Pazour; 1:2,000 for WB; 1:200 for immunofluorescence (IF)], anti-Ac-α-tubulin and anti-γ-tubulin (Sigma; 1:1,000 for IF), anti-vimentin and anti-E-cadherin (Cell Signaling Technology; 1:1,000 for WB; 1:100 for IF), anti-Flag (Abcam; 1:6,000 for WB; 1:600 for IF), anti-Myc (Earthox; 1:6,000 for WB; 1:600 for IF), anti-Numb (Proteintech; 1:1,000 for WB); anti-Strep tag (GenScript; 1:3,000 for WB), anti-mCherry (Earthox; 1:2,500 for WB); anti-GFP (Roche; 1:1,000 for WB); anti-GMAP210 (Novus; 1:100 for IF); and anti-golgin97 (ebioscience; 1 μg/mL for IF). Protein or fixed samples were incubated with primary antibodies overnight at 4°C or for 1 h at room temperature. Secondary antibodies used included horseradish peroxidase (HRP) against mouse or rabbit IgG (Abcam), HRP-conjugated streptavidin (Invitrogen), Alexa Fluor 488- and 555-labeled secondary antibodies (Invitrogen), and Alexa Fluor 568-conjugated streptavidin (Invitrogen). Other staining reagents included DAPI (4′,6-diamidino-2-phenylindole) (Beyotime), Golgi Tracker (Beyotime), and rhodamine phalloidin (Sigma).

### Generation of 4T1 Knockout Cell Lines With the CRISPR/Cas9 System

Single-guide RNAs (sgRNAs) targeting mIFT20 were selected and cloned in the px330-mCherry plasmid. 4T1 cells were transfected with the px330-mCherry plasmid expressing sgRNA of mIFT20. The transduced red fluorescent protein-positive cells were selected via FACS after 36–48 h transfection. Monoclonal cells were separated by placing one cell per well in the FACS system and screened via PCR for homozygous disruption of targeted alleles. Two different IFT20-knockout cell lines were used in subsequent experiments. Sequences for the sgRNA and the primers used for cloning and screening are listed in Supplementary Table 1.

### RNA Extraction and qPCR

Total RNA was extracted from cells using the TRIzol Reagent (Thermo Fisher Scientific) according to the manufacturer's instructions. For first-strand cDNA synthesis, 1 μg of total RNA was reverse-transcribed by using random primers and a ReverAid RT Reverse Transcription Kit (Thermo Fisher Scientific). qPCR was performed in a 7900HT Fast Real-time PCR system by mixing 2 μL of the synthesized cDNA products with SYBR Green and a primer mix to a final volume of 20 μL (TOYOBO). The mean relative gene expression was normalized to glyceraldehyde 3-phosphate dehydrogenase (*Gapdh*) mRNA using the ΔΔC_t_ method.

### Protein Extracts and Western Blots

Cells were collected, washed with phosphate buffered saline (PBS), and resuspended on ice in radioimmunoprecipitation assay (RIPA) buffer (Beyotime) for 10 min with a protease inhibitor cocktail (Sigma) to prevent the degradation of proteins, and then centrifuged at 12,000 rpm at 4°C for 10 min. Supernatants were collected and protein concentrations of whole-cell lysates were determined using amido black 10B. The cell lysates were boiled in sodium dodecyl sulfate polyacrylamide gel electrophoresis (SDS-PAGE) sample buffer and separated using SDS-PAGE. Then proteins were blotted onto a nitrocellulose membrane, probed with specific antibodies, and visualized with enhanced chemiluminescence (ECL, Millipore) on film. All Western blot experiments were repeated three times.

### BioID Assay

Cells were incubated for 12 h with the transfection complex containing pcDNA3.1(-)-IFT20-BirA*-Myc, and then, the solution was replaced with media supplemented with 50 μM biotin or equivalent amounts of dimethyl sulfoxide (DMSO). Cells were further cultured for 36 h. After three washes with PBS, the cells (for small-scale analysis, <10^7^; for large scale analysis, ~3 × 10^7^ cells) were lysed using a mixture of RIPA buffer and protease inhibitors. Supernatants were incubated with 600 μL Dynabeads (MyOne streptavadin C1; Invitrogen) overnight at 4°C. Subsequent elution steps were performed as previously described (Roux et al., 2012).

### Strep-Tactin Pulldown Assay

After transfecting for 24 h with expression vectors, HEK293T cells were washed with PBS, collected with a cell scraper, and lysed using the lysis buffer (10 mM Tris-HCl pH 7.5, 150 mM NaCl, 0.5 mM EDTA, and 0.5% NP-40) containing a protease inhibitors cocktail for 10 min on ice. Lysates were centrifuged at 12,000 × *g* for 10 min at 4°C, and supernatants were incubated with Strep-Tactin XT suspension resin beads (IBA Lifesciences) packed in columns with gaskets. The beads were washed five times with the wash buffer (100 mM Tris-HCl pH 8.0, 150 mM NaCl, and 1 mM EDTA), and the bead-bound proteins (pulldown products) were eluted using the elution buffer (100 mM Tris-HCl pH 8.0, 150 mM NaCl, 1 mM EDTA, and 50 mM biotin) and analyzed by Western blots.

### Immunofluorescence Microscopy and Image Acquisition

Cells were seeded onto coverslips and cultured in complete medium, medium without serum for starvation, or medium with 50 μM biotin for the BioID assay. When growth reached to 60–70% confluence, the cells were washed twice with PBS, fixed with either 4% paraformaldehyde for 15 min or cold methanol for 5 min, and permeabilized with 1% Triton X-100/PBS for 10 min. For F-actin staining, the fixed cells were incubated with rhodamine-phalloidin. For IF staining, the cells were first incubated with primary antibodies diluted in 0.3% Triton X-100, 3% bovine serum albumin (BSA), and 0.1% NaN_3_ in PBS at 4°C overnight. After thorough washing with PBS, the cells were incubated for 1 h with Alexa-Fluor-488- and Alexa-Fluor-555-labeled secondary antibodies (Thermo Fisher Scientific) in 1% BSA and 10 μg/mL DAPI in PBS at room temperature. All immunostaining experiments were performed two or three times.

For fluorescent images, confocal microscopy was performed using a Leica TCS SP8 with a 63× oil objective. The image format was 1,024 × 1,024 pixels using an one Airy unit (AU) pinhole and processed with LAS AF Lite software. The z-axis series of optical sections were performed at 0.8 μm-thick sections. Except for the third line images of **Figure 6D** with a “max” note (z projection), the other images are showed in optical sections. 4T1 cells co-expressing fluorescently-tagged IFT20 and Rab were quantitatively analyzed for colocalization using images of whole cells from three different optical sections (*n* = 21 cells). Image J software (National Institute of Health) was used to determine the Pearson's coefficient, a measurement representing the percentage of pixels from fluorescent IFT20 that overlap with pixels of fluorescent Rab.

### Live Cell Imaging

4T1 cells were seeded onto glass-bottom dishes and allowed to grow to 60% confluence. Next, pEGFP-N1-IFT20 was transfected into 4T1 cells. After 24 h of transfection, the medium was changed to a phenol red-free version of the growth medium, and the cells were visualized with the DeltaVision Elite imaging system (Applied Precision) that contained a stage and objective heater to maintain the cells at 5% CO_2_ and 37°C. Cells with IFT20-EGFP signals were identified and then photographed at 1 s intervals. The images were deconvolved and then converted to a movie displayed at five frames per second with Softworx software (Applied Precision).

### Wound-Healing Assay

The cells were seeded into six-well tissue culture plates and allowed to grow to 90–100% confluence in complete medium. A wound was created by scraping the confluent cell cultures with a 10 μL pipette tip. The floating cells were carefully removed before the medium containing 2% FBS was added. The cells were incubated at 37°C for 12 h. The wound healing process was monitored under an inverted light microscope (Nikon). All wound-healing assays were performed three times.

### Transwell Assay

This cell migration assay was performed by using a Transwell Assay Chamber (PET track-etched membrane; Corning). The cells maintained in serum-free DMEM were seeded in the top chamber and DMEM supplemented with 10% FBS was added to the bottom chamber. Cells can migrate through the transwell membrane. After incubation for 24 h at 37°C, the cells on the top side of the membranes were removed using cotton swabs, whereas those on the bottom side were fixed and stained with crystal violet. Six randomly selected fields per well were photographed, and the number of migrated cells was counted. The migration index of the experimental cells was calculated with the following formula: migration index (%) = (number of cells that migrated through the membrane in the experimental group)/(number of cells that migrated through the membrane in the control group) × 100. All Transwell assays were performed three times.

### Cell Proliferation Assays

Cells were seeded in triplicate in a 96-well plate at ~1,000 cells per well and cultured for 4 d, and cell proliferation was assessed by using the MTS [3-(4,5-dimethylthiazol-2-yl)-5-(3-carboxymethoxyphenyl)-2-(4-sulfophenyl)-2H-tetrazolium] assay according to the manufacturer's protocols (CellTiter 96^®^ AQueous One Solution Cell Proliferation Assay kit; Promega). Briefly, complete DMEM (100 μL) was supplemented with MTS solution 20 μL/well, incubated for 1–4 h, and then the absorbance was recorded at 490 nm with a 96-well plate reader (BioTek). For the colony formation assay, cells were plated in a 6 cm dish at a density of 500 cells per well and cultured for 7 d before being stained with 0.5% crystal violet. The colonies with more than 50 cells were manually counted. All plate colony formation and MTS assays were performed three times.

### Statistical Analysis

Two-tailed Student's *t*-tests were performed to calculate *P*-values unless specified otherwise. All of the experiments were independently performed in triplicate. Error bars represent standard deviations (S.D.). *P*-values ≤0.05 were considered to be statistically significant.

## Results

### The Expression Level of IFT20 Negatively Correlates With the Malignancy of Breast Cancer Cells

**Figure 1:**
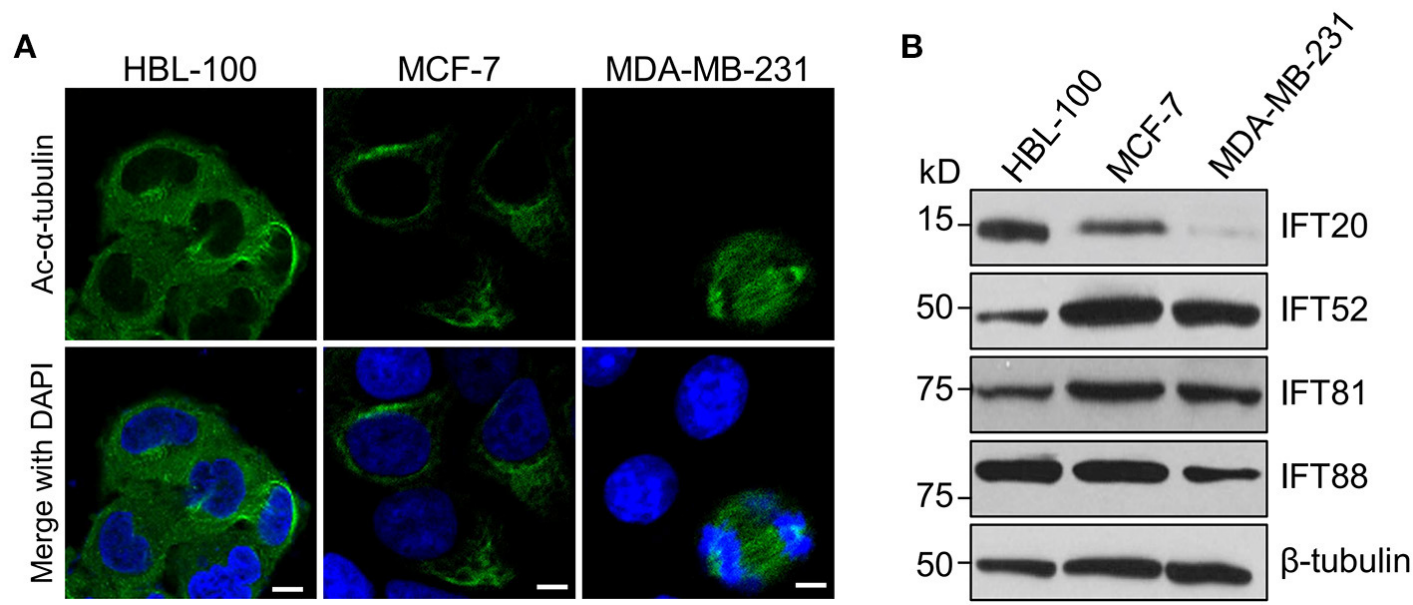
The expression level of IFT20 negatively correlates with the malignancy of breast cancer cells. **(A)** Representative immunostaining of Ac-α-tubulin (green) in HBL-100, MCF-7, and MDA-MB-231 cells from two independent experiments showed that no cilia were formed in these three cell lines when treated with serum starvation for 36 h; Ac-α-tubulin represents acetylated-α-tubulin as the primary cilia marker (green); the nucleus is stained by DAPI (blue). Scale bar, 10 μm. **(B)** Western blots of cell lysates from HBL-100, MCF-7, and MDA-MB-231 cells probed with antibodies raised against IFT20, IFT52, IFT81, and IFT88. β-tubulin was used as the loading control.

To determine the expression of IFT20 in breast cancer cells, two human breast cancer cell lines, MCF-7 and MDA-MB-231, as well as one immortalized human breast epithelial cell line, HBL-100, were chosen for the analyzes (Soule et al., 1973; Cailleau et al., 1974; Chandrasekaran and Davidson, 1979). After these three cell lines were treated with serum starvation for 36 h or not, we stained these samples with antibodies against Ac-α-tubulin, which is the main component of ciliary axonemes and widely used as a marker for cilia (Follit et al., 2006). Immunofluorescence analyzes indicated that no cilia were observed in these three cell lines, although the mitotic spindles and stabilized cytoplasmic microtubules around the nucleus were recognized by this antibody (Figure 1A). Therefore, these three cell lines were suitable for exploring the cilia-independent functions of IFT subunits.

MDA-MB-231 cells have been reported to be more malignant than MCF-7 cells (Silva et al., 2016). Therefore, we compared the expression levels of several IFT subunits between the normal breast epithelial cell line and the breast cancer cells with different malignancy potentials. Western blot analyzes showed that the expression level of IFT20 in HBL-100 cells was higher than that in MCF-7 cells, and the expression of IFT20 was barely detectable in MDA-MB-231 cells (Figure 1B and Supplementary Figure 1A). However, the expression of IFT52 and IFT81 was upregulated in the breast cancer cells, and the expression of IFT88 showed an undetectable difference between HBL-100 and MCF-7 cells (Figure 1B and Supplementary Figures 1B–D). The consistent downregulation of IFT20 in the breast cancer cells with a manner corresponding to the malignancy potential suggests that the expression of IFT20 may be negatively correlated with breast cancer progression; this association was independent of both cilia and the classical IFT complex.

### Knockout of IFT20 Induces Lamellipodia Formation and Epithelial Mesenchymal Transitions (EMTs)

**Figure 2:**
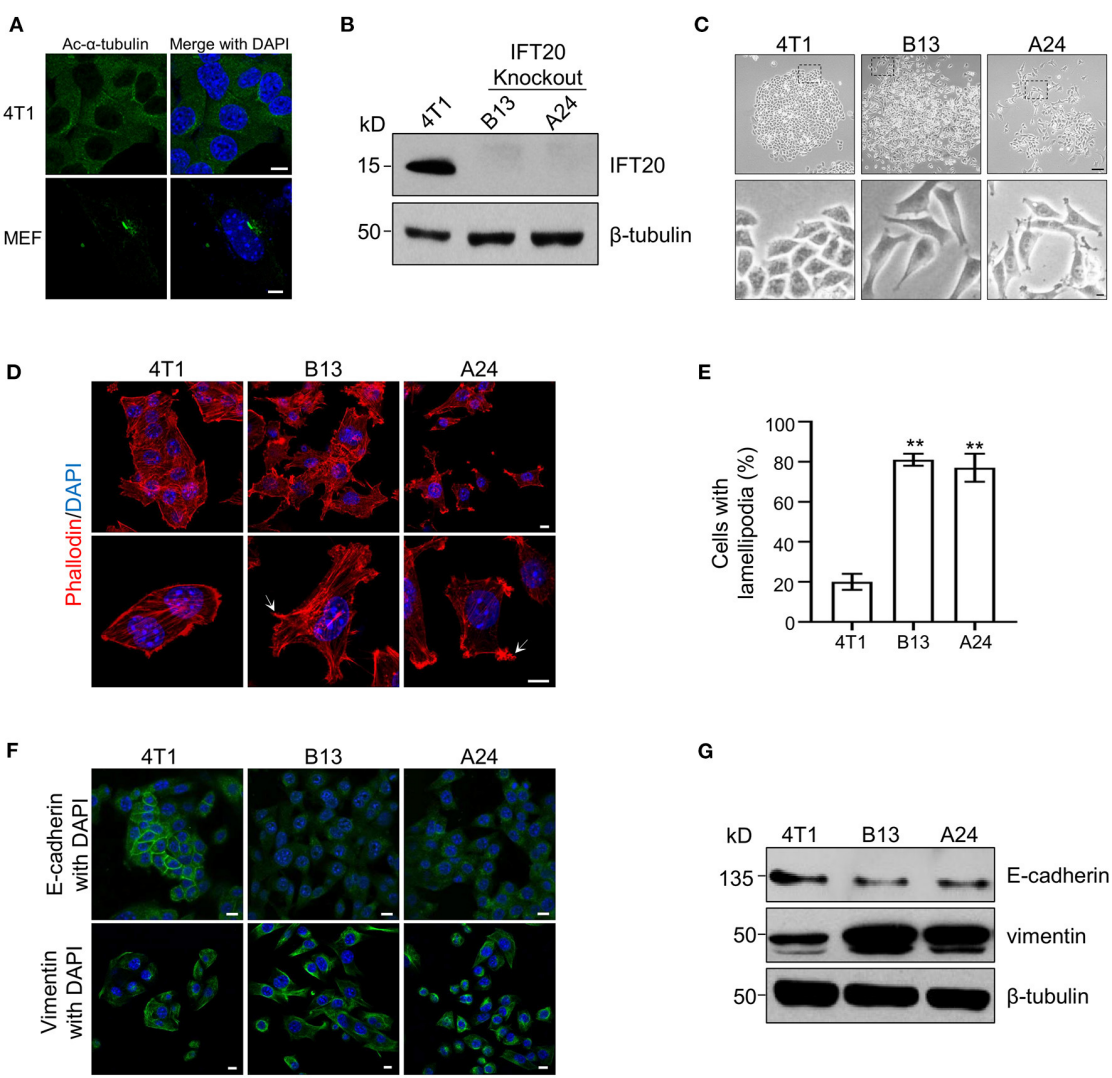
Loss of IFT20 in mouse breast cancer cells 4T1 induces lamellipodia formation and epithelial mesenchymal transitions (EMTs). **(A)** Representative immunostaining of 4T1 and mouse embryo fibroblast (MEF) cells with Ac-α-tubulin antibodies showed that no cilia were formed in 4T1 cells, unlike cilia-positive MEF cells, when treated with serum starvation for 36 h. **(B)** Western blots of cell lysates from 4T1 and IFT20-knockout mutants (B13 and A24) probed with IFT20 antibodies. **(C)** Phase-contrast photographs of 4T1 and IFT20-KO cells showed that loss of IFT20 resulted in cell morphology changes from a cobblestone appearance into spindle-like shapes. The lower panels showed the higher magnification photographs of the insets in the top panels, respectively. **(D)** Representative fluorescent images of F-actin stained with rhodamine B-conjugated phalloidin showed that the loss of IFT20 induced the formation of more lamellipodia. The white arrows indicate the lamellipodia. The nucleus is stained by DAPI. **(E)** Quantification of the cells with lamellipodia in 30 randomly selected cells in each group of **(D)**. Results are representative of three independent experiments. **(F)** Representative immunostaining images of 4T1 and IFT20-KO cells stained with the epithelial marker (E-cadherin) and mesenchymal marker (vimentin) showed that the loss of IFT20 induced EMTs. **(G)** Western blots of cell lysates from 4T1 and IFT20-KO cells probed with epithelial and mesenchymal markers showed that the loss of IFT20 resulted in the down-regulation of E-cadherin but the up-regulation of vimentin. β-tubulin was used as the loading control. All immunostaining experiments were performed three times. The nucleus is stained by DAPI (blue). Scale bar, 5 μm **(A)**; 50 μm (**C** top panel); 10 μm (**C** lower panel, **D,F**). Error bars represent standard deviations. The *p*-values indicated were calculated by Student's *t*-tests (unpaired). n.s. (not significant) *p* > 0.05; *p ≤ 0.05; **p ≤ 0.01.

To elucidate the function of IFT20 in breast cancer cell lines, the mouse breast cancer epithelial cell line, 4T1, was used since the expression level of IFT20 in 4T1 cells was comparable to that in ciliated MEF cells (Supplementary Figure 2A) and no cilia were formed in 4T1 cells treated with or without serum starvation for 36 h (Figure 2A and Supplementary Figure 2B). Using the CRISPR/Cas9 system (Supplementary Figure 2C), several IFT20-KO monoclonal cell lines were obtained. The genotyping analyzes showed that in the two IFT20-KO cell lines (B13 and A24), the translation of IFT20 was terminated early (Supplementary Figure 2D), which was further confirmed by Western blot probed with the IFT20 antibodies (Figure 2B). Interestingly, both B13 and A24 cells can be easily distinguished from 4T1 cells in terms of the morphology. As shown in Figure 2C, 4T1 cells formed a tightly-connected epithelial monolayer with a cobblestone-like appearance. In contrast, B13 and A24 cells did not form a cobblestone-like layer but displayed an elongated, spindle-like, fibroblastic morphology. Occasionally IFT20-KO cells even formed a network-like lattice with long and thin membrane extensions, which indicated the loss of contact inhibition. The actin filaments stained using rhodamine B-conjugated phalloidin revealed that IFT20-KO cells formed more actin bundles under the plasma membrane and more lamellipodia compared with those in 4T1 cells (Figures 2D,E). The change in cell morphology and formation of lamellipodia strongly indicate that loss of IFT20 might induce an EMT, an important early step in the conversion of a tumor into a migratory population capable of undergoing systemic metastasis that occurs by losing epithelial characteristics and acquiring mesenchymal properties (Thiery, 2002).

To address this hypothesis, we examined the expression of epithelial and mesenchymal markers, such as E-cadherin and vimentin, respectively, via Western blots and immunostaining. As shown in Figure 2G, the expression level of E-cadherin was significantly reduced, whereas the expression of vimentin was dramatically increased in IFT20-KO cells compared with those in 4T1 cells (Supplementary Figures 2E,F). Consistent with the Western blots results, up-regulated vimentin and down-regulated E-cadherin were also observed in B13 and A24 cells by immunostaining (Figure 2F). In 4T1 cells, E-cadherin was distributed along the plasma membrane at cell-cell borders, whereas the adhesion junction localization of E-cadherin was lost in B13 and A24 cells. In addition, vimentin showed substantial cytoplasmic staining in B13 and A24 cells. Collectively, we concluded that loss of IFT20 caused active lamellipodia formation and induced an EMT in breast cancer cells.

### Loss of IFT20 Enhances the Migration of Breast Cancer Cells

**Figure 3:**
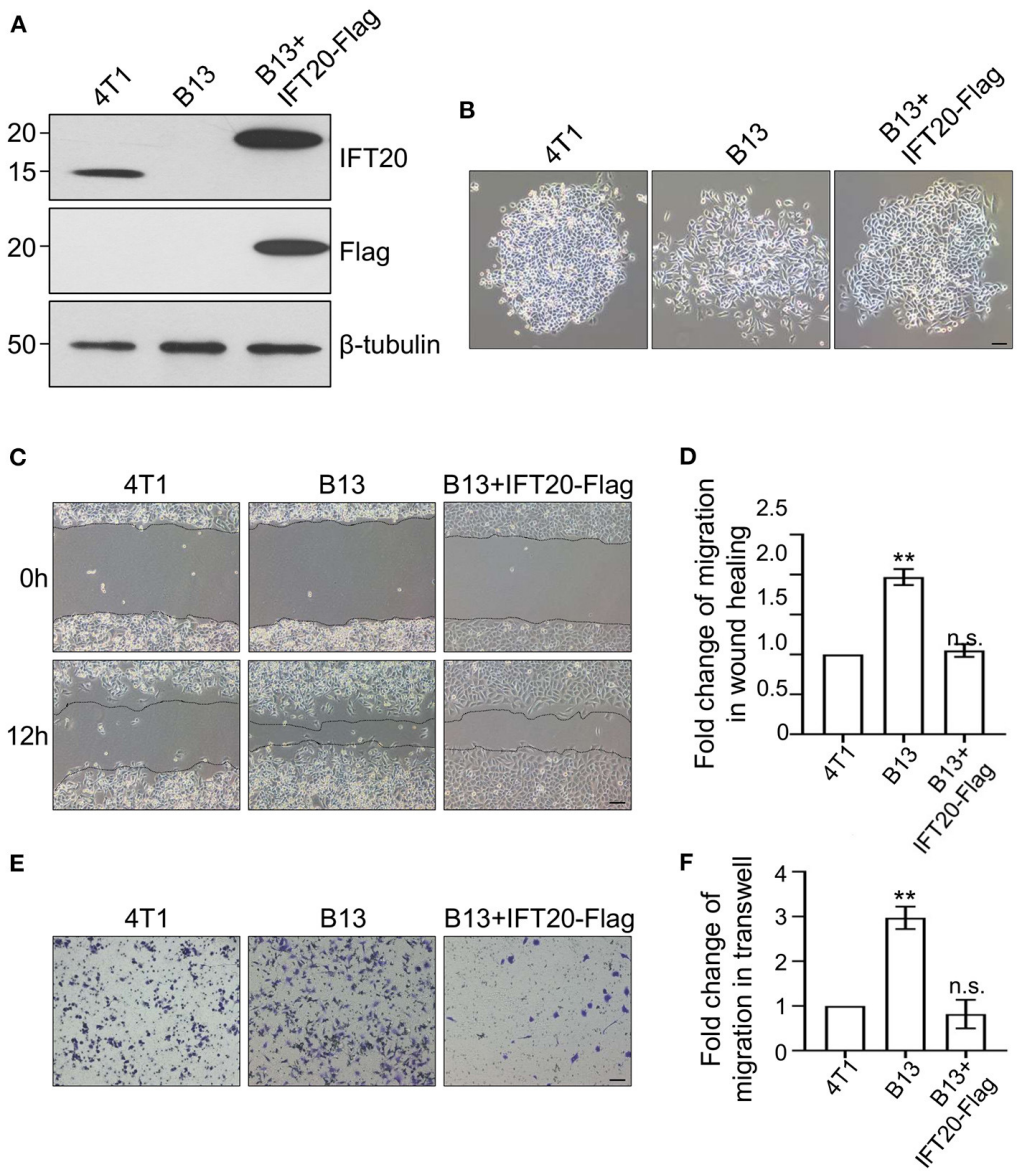
Loss of IFT20 enhances the migration of breast cancer cells. **(A)** Western blots of lysates from 4T1, B13 (IFT20-KO), and B13+IFT20-Flag cells (B13 cells stably expressing IFT20-Flag) probed with IFT20 antibodies. β-tubulin was used as the loading control. **(B)** Phase-contrast images of 4T1, B13, and B13+IFT20-Flag cells. **(C)** Representative pictures of cell migration in 4T1, B13, and B13+IFT20-Flag cells as evaluated by the wound healing assay. **(D)** Quantification of the wound healing distance in **(C)** after 12h showed that the loss of IFT20 enhanced horizontal migration. *n* = 24 fields of view in three independent experiments. **(E)** Representative pictures of cell migration in 4T1, B13, and B13+IFT20-Flag cells as evaluated by the transwell assay. **(F)** Quantification of migrated cell numbers in **(E)** showed that the loss of IFT20 significantly enhanced cell migration. *n* = 18 fields of view in three independent experiments. Represented data are the mean ± SD in three biological replicates. n.s. (not significant) *p* > 0.05; *p ≤ 0.05; **p ≤ 0.01. Scale bar, 50 μm.

To confirm the accuracy of the CRISPR/Cas9 knockout, we reconstituted IFT20 expression in B13 cells through a lentivirus expression system and monoclonal cell lines were screened using puromycin. The re-expression of IFT20 in one of monoclonal cell lines, named B13+IFT20-Flag, was validated via Western blot, and the fusion protein was recognized by both IFT20 and Flag antibodies (Figure 3A). As expected, the altered cell morphology owing to the deletion of IFT20 was rescued in B13+IFT20-Flag cells (Figure 3B).

Owing to the morphological change observed in B13 and A24 cells, loose cell-cell junctions and active lamellipodia formation, are preconditions for cell migration. Therefore, we investigated whether loss of IFT20 influenced the migratory ability of breast cancer cells. As shown in Figure 3C, B13 cells exhibited significantly faster wound closure than 4T1 cells, and the migration distance of B13 cells was approximately twice that of 4T1 cells after 12 h in the monolayer wound healing assay (Figure 3D). B13+IFT20-Flag cells showed no evident migration difference compared with 4T1 cells, indicating that loss of IFT20 markedly enhanced horizontal migration. Similar results were also obtained in the Transwell assay, where the migrated cell number from the upper chamber in B13 cells was twice compared with 4T1 or B13+IFT20-Flag cells (Figures 3E,F). All data indicated that loss of IFT20 enhanced the migration of breast cancer cells.

As highly proliferative cells may influence the results from wound healing and Transwell assays by increasing the number of cells, to rule out this possibility, we determined the effects of IFT20 deficiency on cell proliferation using plate clone formation and MTS assays. In the plate clone assay, the number of clones in B13 and A24 cells was significantly fewer and the shape of clones was smaller than that in 4T1 cells (Supplementary Figures 3A,B). Additionally, in the MTS assay, B13 and A24 also showed delayed proliferation (Supplementary Figure 3C). These results indicated that depleting IFT20 in 4T1 cells exerted an inhibitory effect on cell proliferation. The enhanced migration was not due to excessive proliferation, which was similar to the reported early behavior for breast cancer cell metastasis, including inhibition of proliferation and stimulation of migration (Kedrin et al., 2008). Taken together, IFT20 might participate in the early metastasis of breast cancer cells, and the deletion of IFT20 enhanced the cell migratory potential.

### IFT20 Localizes at the Trans-Golgi/TGN and in the Post-Golgi Vesicles

**Figure 4:**
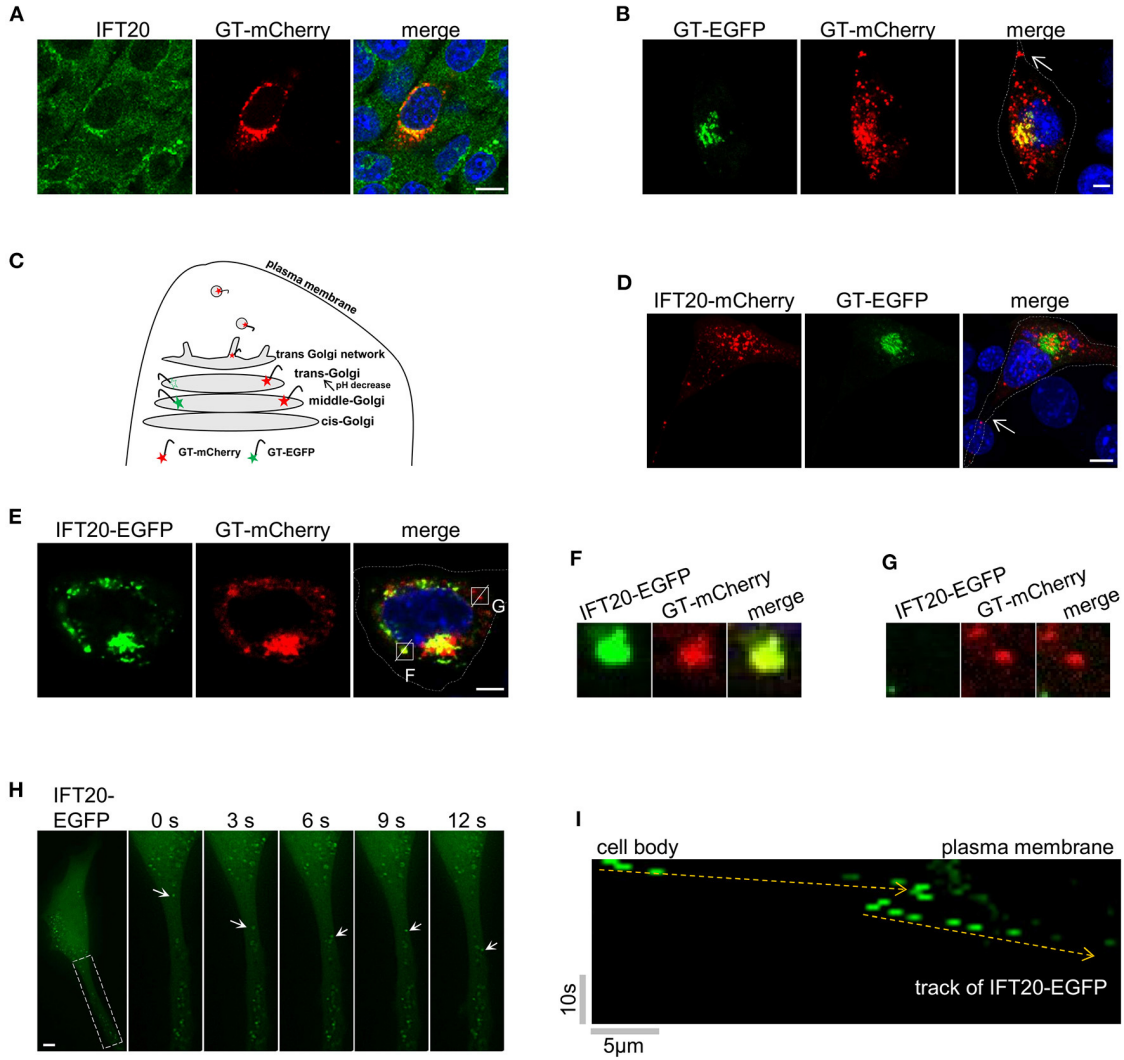
IFT20 localizes at the trans-Golgi, TGN, and in the post-Golgi vesicles. **(A)** Representative fluorescent images of 4T1 cells expressing GT-mCherry and stained with IFT20 antibodies showed that IFT20 localized at the Golgi. **(B)** Representative fluorescent images of 4T1 cells expressing GT-EGFP and GT-mCherry showed that GT-EGFP labels the middle-Golgi and GT-mCherry labels the middle-Golgi, trans-Golgi, and some dot signals; arrow indicates the dot signal of GT-mCherry in cell protrusion. **(C)** Schematic illustrating the markers used to distinguish the middle-Golgi and trans-Golgi based on their sensitivity to pH. EGFP and mCherry fluorophores were directed to the lumen of the middle-Golgi and trans-Golgi by fusing with the Golgi targeting domain of β-1,4-galactosyltransferase (GT). The fluorescence of EGFP disappeared as a result of the lower pH at the lumen of the trans-Golgi compared with middle-Golgi, whereas mCherry fluorescence was stable. In 4T1 cells, GT-mCherry was also found at the TGN and in the post-Golgi vesicles. **(D)** Representative fluorescent images of 4T1 cells expressing GT-EGFP and IFT20-mCherry showed that IFT20 did not localize at the middle-Golgi; arrow indicates the dot signal of IFT20-mCherry in cell protrusion. **(E)** Representative fluorescent images of 4T1 cells expressing IFT20-EGFP and GT-mCherry showed that IFT20 colocalized with GT-mCherry not only at the trans-Golgi/TGN, but also in the post-Golgi vesicles. **(F,G)** Magnified views of the indicated insets in **(E)** showing complete colocalization **(F)** or no-colocalization **(G)** between IFT20-EGFP and GT-mCherry post-Golgi vesicles. The white dotted lines mark the entire cell profile. **(H)** Representative time-lapse images showing dynamic transport of IFT20-EGFP in cell protrusion. S, second. **(I)** The kymograph from a representative dot signal of IFT20-EGFP indicated by the arrows in **(H)**. Yellow dashed lines indicate the track of IFT20-EGFP. Horizontal scale bar is 5 μm; vertical bar is 10 s. All fluorescent experiments were performed two **(A,H)** or three times **(B,D,E)**. The nucleus was stained by DAPI (blue). Scale bar, 5 μm.

To further dissect the role of IFT20 in 4T1 cells, the localization of IFT20 was determined by immunostaining with IFT20 antibodies and the Golgi marker plasmid GT-mCherry. An evident overlap between IFT20 and GT-mCherry was observed in Figure 4A, which suggests that IFT20 localized at the Golgi in 4T1 cells. To determine the exact localization of IFT20 at the cis-Golgi, middle-Golgi, or trans-Golgi, two fluorescent proteins were used to selectively label these Golgi sub-regions.

Specifically, GT-mCherry and GT-EGFP, which contained the same Golgi-localized sequence (GT) but were coupled with different pH-sensitive fluorescent proteins, were exploited to distinguish the middle-Golgi and trans-Golgi, and this technique was feasible because of the gradually reduced pH gradient from the cis- to trans-side lumen of the Golgi (Mellman and Simons, 1992). The GT motif was a N-terminal Golgi-targeted sequence of β-1,4-galactosyltransferase 1 (B4GALT1), which displayed a middle- and trans-Golgi distribution (Shaper et al., 1988). When GT-EGFP was expressed in 4T1 cells, signals from the fusion proteins were only observed at the middle-Golgi. A signal in the trans-Golgi was not observed because of the sensitivity of EGFP to the acidic environment in the trans-Golgi lumen. When GT-mCherry was expressed, the fluorescent signal was observed at both the middle- and trans-Golgi because mCherry is not sensitive to acidic environments. As shown in Figure 4B, 4T1 cells expressing both GT-mCherry and GT-EGFP showed a limited merged yellow signal at the middle-Golgi and a separate red signal at the trans-Golgi. The EGFP signal of GT-EGFP-mCherry was also only detected at the middle-Golgi (Supplementary Figure 4A).

When 4T1 cells expressing GT-EGFP were stained by the red Golgi tracker, the signal of GT-EGFP was restricted to a partial reticulum-like structure, accompanying the colocalization with the red Golgi Tracker (Supplementary Figure 4B). Meanwhile, GT-EGFP and GT-mCherry both showed a near but not overlapping distribution with the cis-Golgi marker GMAP210 (Supplementary Figures 4C,D). Collectively, these data indicated that GT-EGFP was a reliable marker for the middle-Golgi and that GT-mCherry could be used to monitor the localization at both the middle- and trans-Golgi, where the pH value of the lumenal environment of the trans-Golgi in 4T1 cells was between 4.5 and 6.0 based on the pKa of EGFP and mCherry. Notably, in addition to the middle- and trans-Golgi localization, we also observed dot signals of GT-mCherry in cell protrusions (arrow in Figure 4B). As an unusual glycosyltransferase, particularly in defined cell types, such as fibroblasts and HepG2 cells, B4GALT1 follows the Golgi-secretory pathway from the trans-most cisterna, TGN, to the plasma membrane (Shur, 1993; Schaub et al., 2006). Additionally, GT-mCherry partially colocalized with golgin97, one marker of trans-Golgi network in HeLa cells (Supplementary Figure 4E). Therefore, the GT-mCherry-labeled dots were mostly a certain type of TGN-derived vesicles (Figure 4C).

Then, IFT20-mCherry and GT-EGFP were co-expressed in 4T1 cells. As shown in Figure 4D, GT-EGFP showed cisternae-like and compact signals around the nucleus. IFT20-mCherry showed no colocalization with GT-EGFP but was perfectly juxtaposed to it, demonstrating that IFT20 did not localize at the middle-Golgi. Notably, some dot signals of IFT20-mCherry were observed in cellular protrusions as well (arrow in Figure 4D). Next, we compared the distribution pattern of IFT20-EGFP and GT-mCherry. The perinuclear distribution of GT-mCherry was large and compact, along with some smaller dot signals, while IFT20-EGFP colocalized with GT-mCherry at this stack region (Figure 4E). Since IFT20 had no transmembrane motif and did not enter into the lumen of the Golgi, the lumenal environment of the Golgi had no effect on the fluorescence of IFT20-EGFP. In addition to their predominant Golgi colocalization, some (but not all) of the dot signals of these two proteins also overlapped (Figures 4F,G). To determine the dynamics of IFT20, a live-cell imaging time-lapse at 1 s intervals was used to observe the movement of IFT20-EGFP. The movies showed anterograde transport of IFT20-associated vesicles from the cell body to cell projections (Figures 4H,I and Supplementary Video 1). Taken together, IFT20 localized at the trans-Golgi/TGN and may transport specific post-Golgi vesicles to the plasma membrane in breast cancer cells.

### IFT20 Mediates the Vesicle Transport From the TGN to the Plasma Membrane

The TGN is a tubular network that sorts proteins toward different destinations, such as the plasma membrane, early endosome, recycling endosome, late endosome, or earlier Golgi compartments, in which an unique set of Rab proteins and their effectors coordinate consecutive stages to mediate specific vesicle transport pathways. Therefore, highly compartmentalized Rabs are suitable markers to determine and label the specificity of transport pathways. Six classes of Rab proteins were selected to determine the specificity of IFT20-associated vesicle transport pathways, and brief descriptions of these Rab GTPases are provided in Supplementary Figure 5A (Stenmark, 2009).

**Figure 5:**
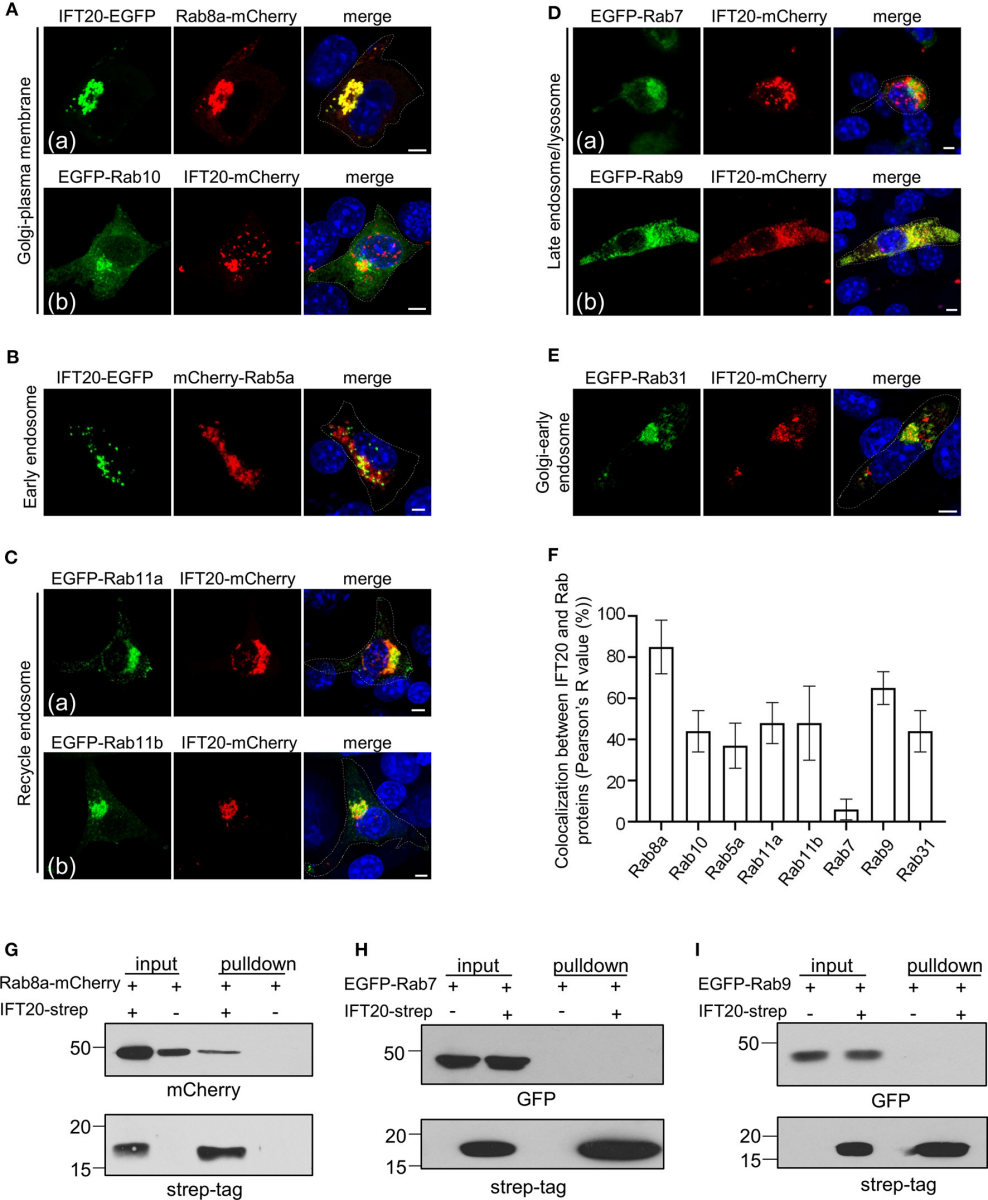
IFT20 mediates the vesicle transport from the TGN to the plasma membrane. **(A)** Fluorescent images of 4T1 cells co-expressing the Golgi-to-plasma membrane Rab markers Rab8a-mCherry **(a)** or EGFP-Rab10 **(b)** with IFT20-EGFP/mCherry. **(B)** The localization pattern of the early endosome Rab marker mCherry-Rab5a with IFT20-EGFP in 4T1 cells. **(C)** Representative fluorescent images of 4T1 cells co-expressing the recycle endosome Rab markers EGFP-Rab11a **(a)** and EGFP-Rab11b **(b)** with IFT20-mCherry. **(D)** The localization of the late endosome-associated Rab markers EGFP-Rab7 **(a)** or EGFP-Rab9 **(b)** with IFT20-mCherry in 4T1 cells. **(E)** Fluorescent images of 4T1 cells co-expressing the Golgi-to-early endosome Rab marker EGFP-Rab31 with IFT20-mCherry. **(F)** The localization quantification (Pearson's R value) of IFT20 and Rab proteins in **(A–E)**. Data are recorded in Supplementary Table 2 and represented as the mean ± SD with three different optical sections on 21 cells co-expressing fluorescent proteins in three independent experiments. **(G–I)** Strep-pulldown assay of IFT20-Strep and Rab8a-mCherry **(G)**, EGFP-Rab7 **(H)**, or EGFP-Rab9 **(I)**. HEK293T cells were transfected with plasmids that express Rab8a-mCherry **(G)**, EGFP-Rab7 **(H)**, or EGFP-Rab9 **(I)** combined with or without IFT20-Strep expression plasmid. After 24 h, the cells were lysed and centrifuged. The supernatants (input) were incubated with Strep-Tactin beads, and the proteins bound to the beads (pulldown) were analyzed by Western blot using anti-Strep tag or anti-mCherry/EGFP antibodies. The white dotted lines mark the entire cell profile. The nucleus is stained by DAPI (blue). Scale bar, 5 μm.

Rab8a and Rab10 mainly participated in the Golgi-to-plasma membrane transport pathways. In 4T1 cells expressing both IFT20-EGFP and Rab8a-mCherry, IFT20-EGFP was highly enriched on Rab8a-positive structures not only at the perinuclear TGN, but also within intracellular vesicles, with a colocalization indicator (Pearson's R value) >80% [Figures 5A(a),F]. To show the weaker cytosolic vesicle signal, the detectors were enhanced, which might cause some signal to saturate. To rule out an artifact owing to these saturated signals at the perinuclear regions, multiple images taken along the z-axis were used to evaluate the spatial relationship of IFT20 and Rab proteins (as represented in Supplementary Figure 5B). In contrast to Rab8a, IFT20 showed less perinuclear co-distribution with Rab10 [Figure 5A(b)]. Based on these two results, we theorized that IFT20 may participate in the vesicle transport targeting to the plasma membrane, which was selectively associated with Rab8a.

Besides exocytosis vesicular pathways, we also determined whether IFT20 was involved in endocytic-associated (plasma membrane-to-endosome or endosome-to-TGN) pathways. Rab5a, an early endosome marker, showed a slight colocalization with IFT20 at the perinuclear region. The vesicle-like signal of IFT20-EGFP near the plasma membrane was adjacent to but excluded from the structure positive for Rab5a (Figure 5B). IFT20 also partially colocalized with Rab11a or Rab11b-associated recycle endosomes, which typically accumulated around the centrosome and in close vicinity to the Golgi (Figure 5C). Conversely, although lysosomes were also generally concentrated around the perinuclear region (Matteoni and Kreis, 1987), there was no colocalization between IFT20-mCherry and EGFP-Rab7, a late endosome/lysosome marker [Figure 5D(a)].

Rab31 is responsible for the vesicle transport from the TGN to the early endosome, while Rab9 is responsible for the vesicle recycling from the late endosome to the TGN (Stenmark, 2009). The bulk of EGFP-Rab9 colocalized with IFT20-mCherry [Figure 5D(b)], whose colocalization indicator was more than 70% (Figure 5F). On the other hand, we did not observe vesicle-like colocalization of Rab31 and IFT20 except for the TGN partial colocalization or neighboring distribution at the cytoplasm (Figure 5E). These results suggested that IFT20 might engage in retrograde transport from the late endosome to the TGN.

To further evaluate their potential interaction, we next performed Strep-Tactin pulldown assay with lysates from HEK293T cells that overexpressed strep-tagged IFT20 and EGFP/mCherry-tagged Rab proteins. These pulldowns revealed an association between IFT20-strep and Rab8a-mCherry (Figure 5G). However, interaction was not observed between IFT20-strep and EGFP-Rab7 or -Rab9 (Figures 5H,I).

Collectively, although we readily observed the perinuclear colocalization of IFT20 and several Rab proteins, such as Rab10, Rab11a, Rab11b, and Rab31, there was no observed vesicular colocalization between these Rabs and IFT20. Clearly, IFT20 was particularly present in Rab8a- and Rab9-positive vesicles/structures, demonstrating that IFT20 mostly participated in the plasma membrane-targeted vesicle transport from the TGN and the retrograde transport from the late endosome to the TGN. Moreover, we detected an interaction between IFT20 and Rab8a using Strep-Tactin pulldown assay. Further elucidating the involvement of IFT20 in vesicle trafficking from TGN to plasma membrane will help us to better understand the role of IFT20 in breast cancer cell migration.

### Identification of IFT20 Interactors Using the BioID Method

To further determine the role of IFT20 in breast cancer cells, it was necessary to identify the cargoes transported through the IFT20-associated vesicles. We took advantage of the proximity-dependent BioID and mass spectrometry (MS) to detect the interacting proteins of IFT20. The key component of BioID is BirA*, an engineered enzyme from *Escherichia coli* that promiscuously biotinylates proteins in a proximity-dependent fashion. In mammalian cells, BirA* is diffused throughout the cell, unless it is fused to a target protein with specific localization tendencies. In the presence of exogenous biotin, BirA* pronouncedly biotinylates proteins that are in proximity to BirA* or fusion proteins (Roux et al., 2012).

**Figure 6:**
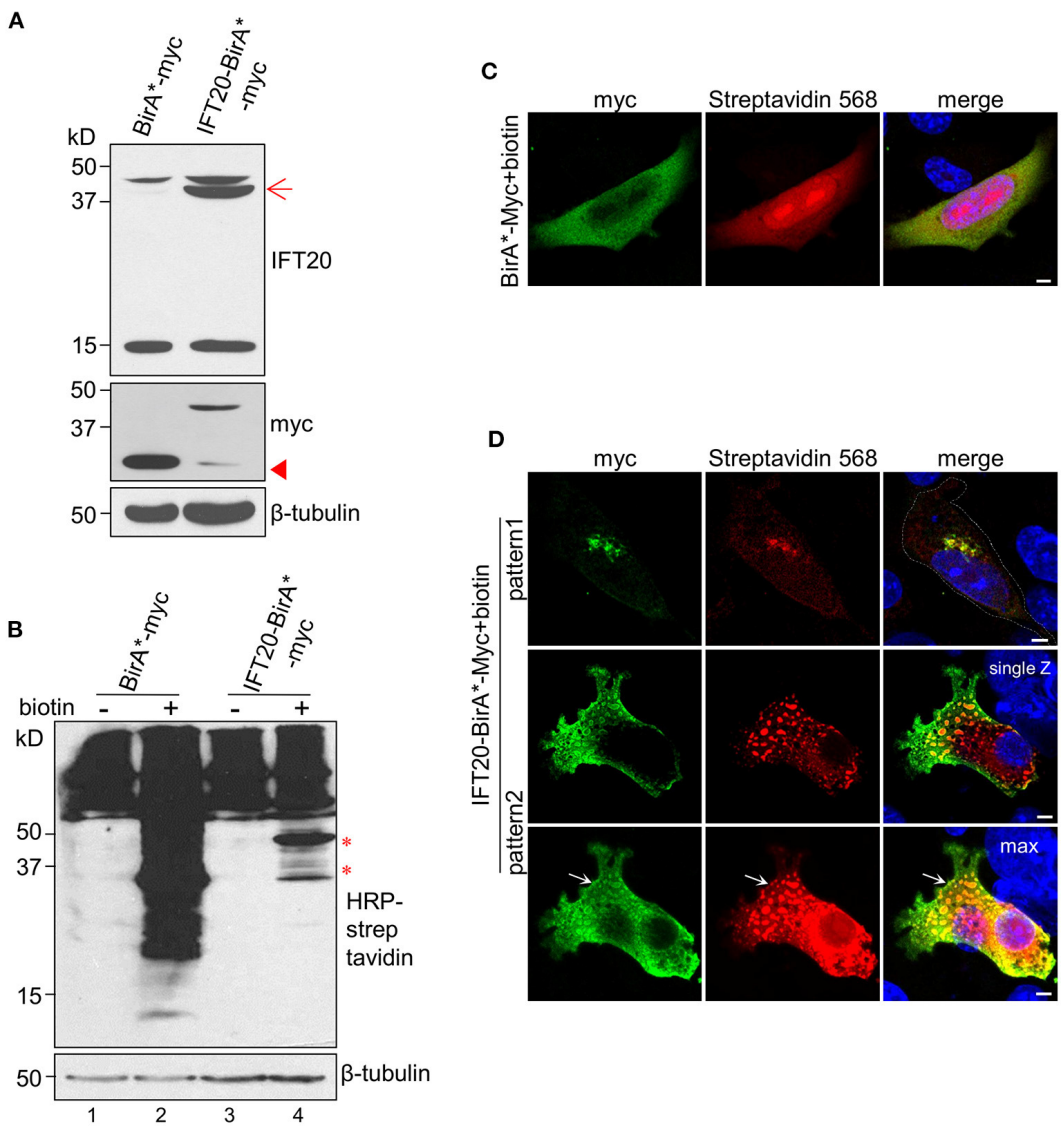
Identification of IFT20 interactors using the BioID method. **(A)** Western blots from lysates of 4T1 cells expressing IFT20-BirA*-Myc or BirA*-Myc probed with IFT20 and Myc antibodies. The arrow points to the anticipated band of IFT20-BirA*-Myc. The arrowhead points to the band of slight truncation of IFT20-BirA*-Myc. **(B)** Western blots from lysates of 4T1 cells expressing IFT20-BirA*-Myc or BirA*-Myc probed with HRP-streptavidin with/without biotin addition; the results showed that the amount of biotinylated proteins increased when biotin was added. The asterisks indicate the specified bands with IFT20-BirA*-Myc in the presence of biotin. The numbers below represent different columns. **(C)** Representative immunofluorescence of 4T1 cells expressing BirA*-Myc in the presence of biotin probed with Myc antibodies to show the cytosol localization of BirA*-Myc and probed with streptavidin 568 to show the biotinylated proteins. **(D)** Representative immunofluorescence images of 4T1 expressing IFT20-BirA*-Myc in the presence of biotin probed with Myc antibodies to show the localization of fusion proteins and probed with streptavidin 568 to show the localization of biotinylated proteins. The top panels show the Golgi localization, while the lower two panels (representing the single Z-stack and max projection with five Z-stacks) show the vesicle-like localization of IFT20-BirA*-Myc and biotinylated proteins. The white dotted lines mark the entire cell profile. The white arrows indicate the vesicle-like distribution near the plasma membrane of IFT20-BirA*-Myc. The z-axis series of optical sections were performed at 0.8 μm-thick sections. The β-tubulin was used as the loading control in the Western blots. All immunostaining experiments were performed two times. The nucleus is stained by DAPI (blue); scale bar, 5 μm.

The main procedures of IFT20-BioID are illustrated in Supplementary Figure 6A. The expression of the fusion protein BirA*-Myc or IFT20-BirA*-Myc was confirmed as shown in Figure 6A. Without biotin addition, the amount of endogenous biotinylated proteins in cells expressing BirA*-Myc or IFT20-BirA*-Myc was similar (Figure 6B, lane 1 and lane 3). When 50 μM biotin was added to the culture medium, massive accumulations of biotinylated proteins were detected in both cell lines. In addition, some bands specifically appeared in the cells expressing IFT20-BirA*-Myc (Figure 6B, lane 4) and might represent the putative IFT20-interacting proteins.

The localizations of BirA*-Myc and IFT20-BirA*-Myc were also compared. BirA*-Myc was randomly distributed in the cytoplasm. Accordingly, proteins biotinylated via BirA*-Myc also showed a diffuse cytoplasmic distribution when biotin was added (Figure 6C). In contrast, IFT20-BirA*-Myc fusion proteins and corresponding biotinylated proteins predominantly localized at the Golgi, similar to the distribution of endogenous IFT20 and IFT20-GFP (Figure 6D, pattern 1). Notably, IFT20-BirA*-Myc fusion proteins also showed vesicle-like localization, in which the Myc antibodies labeled the vesicular boundaries and the streptavidin-568 labeled the intra-vesicular biotinylated proteins. When several z-axis images were projected into one max image, an accumulation of IFT20-associated vesicles at the cell margin was observed (arrow in Figure 6D, pattern 2). Then, the biotinylated proteins were purified using streptavidin-coupled magnetic beads, which can be validated by the obvious enrichment of the Myc signal in the elution of IFT20-BirA*-Myc group, which was not seen in the supernatant (Supplementary Figure 6B). The purified proteins from IFT20-biotin (4T1 cells expressing IFT20-BirA*-Myc with biotin) and another two control groups (parental 4T1 cells and 4T1 cells expressing IFT20-BirA*-Myc with DMSO) were subjected to MS identification (Supplementary Table 3).

The proteins identified in the IFT20-biotin and IFT20-DMSO groups were ranked according to their fold-change values, which were mostly <2 (with one exception for IFT20 itself) (Supplementary Table 4). Proteins unique to IFT20-biotin and not detected in the other two controls were ranked by the spectral counts adjusted to the protein length (Supplementary Table 5) (Roux et al., 2012).

### IFT20 Is Involved in Transporting Numb and Ctnnal1 From the TGN to the Plasma Membrane

**Figure 7:**
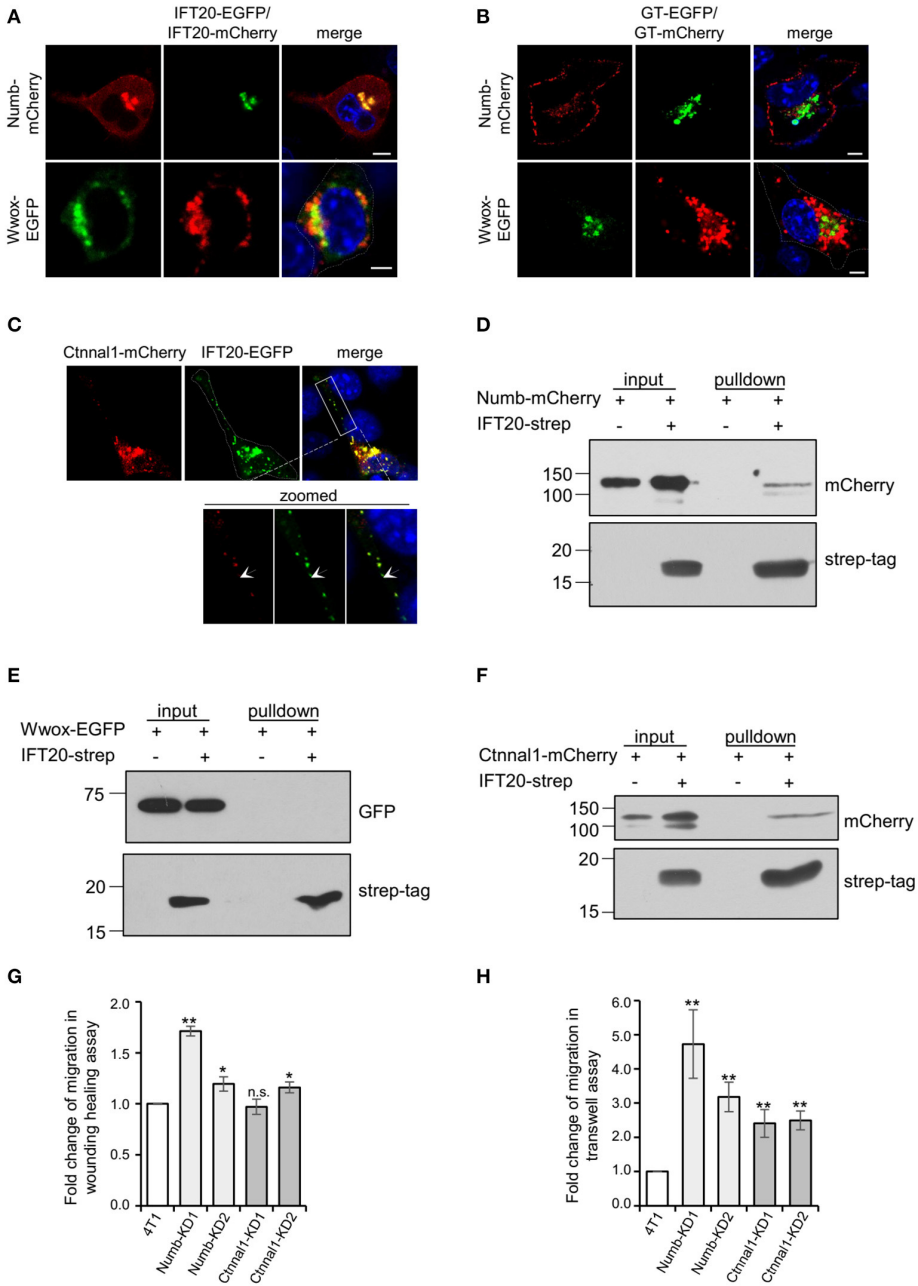
IFT20 is involved in transport Numb and Ctnnal1 from the TGN to the plasma membrane. **(A)** Representative fluorescent images of 4T1 cells co-expressing IFT20-EGFP/mCherry and Numb-mCherry or Wwox-EGFP showing that IFT20 colocalized with Numb and scarcely colocalized with Wwox at the perinuclear region. **(B)** The respective distribution of GT-EGFP/GT-mCherry and Numb-mCherry or Wwox-EGFP showed that the perinuclear localization of Numb was trans-Golgi/TGN and Wwox localized at the cis-Golgi. **(C)** Representative fluorescent images of 4T1 cells co-expressing IFT20-EGFP and Ctnnal1-mCherry showed that IFT20 colocalized with Ctnnal1 not only at the Golgi, but also in the intracellular vesicles. The white dotted line marks the entire cell profile. The white dashed rectangular box marks the zoomed images on the bottom. **(D–F)** Strep-pulldown assay of IFT20-Strep and Numb-mCherry **(D)**, EGFP-Wwox **(E)**, or Ctnnal1-mCherry **(F)**. HEK293T cells were transfected with plasmids that express Numb-mCherry **(D)**, EGFP-Wwox **(E)**, or Ctnnal1-mCherry **(F)** combined with or without IFT20-Strep expression plasmid. After 24 h, the cells were lysed and centrifuged. The supernatants (input) were incubated with Strep-Tactin beads, and the proteins bound to the beads (pulldown) were analyzed by Western blot using anti-Strep tag or anti-mCherry/EGFP antibodies. **(G,H)** Quantification of the migration efficiency in the wound healing assay **(G)** and transwell assay **(H)**; results showed that knockdown of Numb and Ctnnal1 enhanced breast cancer cell migration. All quantifications were carried out in three independent experiments, and data are expressed as the mean ± S.D.; n.s. (not significant) *p* > 0.05; *p ≤ 0.05; **p ≤ 0.01. All fluorescent experiments were performed three times. The nucleus is stained by DAPI (blue); scale bar, 5 μm.

To confirm the interactions between IFT20 and candidate proteins identified in BioID, the localizations of these proteins were studied. A considerable colocalization between IFT20 and Numb was observed at the perinuclear Golgi region (Figure 7A). Numb was also detected in small vesicles around the middle-Golgi, but did not overlap with GT-EGFP, which suggested that Numb was localized at the trans-Golgi/TGN. Moreover, Numb also showed a discrete distribution at the substratum of the plasma membrane (Figures 7A,B), which might be one of its functional sites for the mediation of the endocytosis of Notch1 receptors (Colaluca et al., 2008). Different from Numb, the actual degree of overlap between IFT20-mCherry and Wwox-EGFP was limited, despite the close spatial apposition (Figure 7A). Additionally, Wwox, a WW domain-containing oxidoreductase without a transmembrane domain (Ludes-Meyers et al., 2004), scarcely colocalized with GT-mCherry (Figure 7B), which indicated that Wwox resided at the cis-Golgi.

Ctnnal1 (catenin-alpha-like 1), a cytoskeletal linker protein under the plasma membrane (Park et al., 2002), colocalized with IFT20 not only at the perinuclear Golgi but also in the intracellular vesicles at cell projections (Figure 7C), suggesting that IFT20 was associated with the transport of Ctnnal1 from the Golgi to the plasma membrane. We also found that strep-tagged IFT20 pulled down Ctnnal1-mCherry and Numb-mCherry but not Wwox-EGFP expressed in lysates from HEK293T cells (Figures 7D–F). Given the colocalization and interaction of IFT20 with Numb and Ctnnal1, we generated 4T1 cell lines stably expressing sh-Numb or sh-Ctnnal1 (Supplementary Figures 7A,B). As shown in Figure 7G, Numb-KD1, Numb-KD2, and Ctnnal1-KD2 cells exhibited faster wound closure than that in negative control (NC) cells, indicating that down-regulated Numb and Ctnnal1 enhanced breast cancer cell migration. Similar results were also obtained in the Transwell assay that knockdown of Numb and Ctnnal1 promoted breast cancer cell migration even for Ctnnal1-KD1 cells whose downregulation may not have been as strong as that in the Ctnnal1-KD2 cells (Figure 7H). Therefore, we speculated that IFT20 was involved in transporting Numb and Ctnnal1 from the TGN to the plasma membrane. When IFT20 was depleted, these proteins could not be efficiently transported to the plasma membrane, which promoted breast cancer cell migration.

### Interactions of IFT20 With the F-Actin Associated Protein Tagln2 Regulates the Migration of Breast Cancer Cells

**Figure 8:**
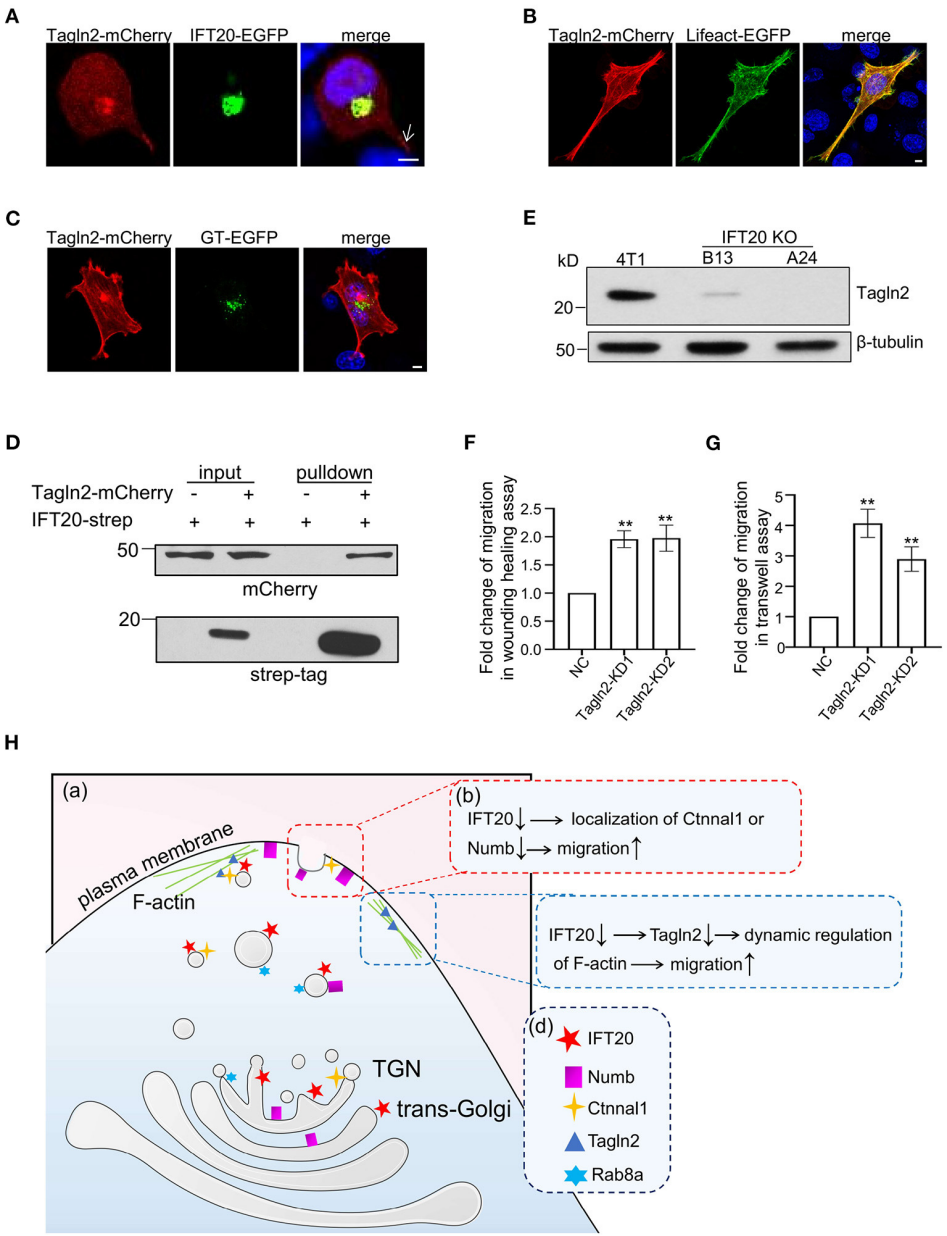
Interactions of IFT20 with the F-actin associated protein Tagln2 regulates the migration of breast cancer cells. **(A)** Representative fluorescent images of 4T1 cells co-expressing IFT20-EGFP and Tagln2-mCherry showed that IFT20 co-localizes with Tagln2. Arrow indicates the signal of Tagln2-mCherry at the cell projection. **(B)** Representative fluorescent images of 4T1 cells expressing Tagln2-mCherry and Lifeact-EGFP showing the F-actin localization of Tagln2. **(C)** Representative fluorescent images of 4T1 cells co-expressing GT-EGFP and Tagln2-mCherry showing the adjacent localization of Tagln2-mCherry to GT-EGFP. **(D)** Strep-pulldown assay of IFT20-Strep and Tagln2-mCherry. HEK293T cells were transfected with plasmids that express Tagln2-mCherry combined with or without IFT20-Strep expression plasmid. After 24 h, the cells were lysed and centrifuged. The supernatants (input) were incubated with Strep-Tactin beads, and the proteins bound to the beads (pulldown) were analyzed by Western blot using anti-Strep tag or anti-mCherry antibodies. **(E)** Western blots of cell lysates from 4T1 and IFT20-knockout mutants (B13 and A24) probed with Tagln2 antibodies showing the downregulation of Tagln2 in IFT20-KO cells. β-tubulin was used as the loading control. **(F,G)** Quantification of the migration efficiency in the wound healing **(F)** and transwell **(G)** assays showed that the downregulation of Tagln2 significantly enhanced cell migration. **(H)** A working model of IFT20-associated vesicles transporting Numb and Ctnnal1 to the plasma membrane in breast cancer cells. **(a)** Trans-Golgi/TGN localized IFT20 is involved in the vesicle transport of Numb and Ctnnal1 from the Golgi to the plasma membrane, which is overlapped with the Rab8a-positive trafficking pathway. **(b)** The amount of Numb and Ctnnal1 decreased at the plasma membrane because of the loss of IFT20, which enhances the migration of 4T1 cells. **(c)** IFT20 interacts with F-actin-associated protein Tagln2; loss of IFT20 causes the downregulation of Tagln2, which enhances the migration of 4T1 cells. **(d)** A key showing the shapes used in the illustration and their corresponding cellular components. All quantitative graphs were constructed with data from three independent experiments and date are expressed as the mean ± S.D.; n.s. (not significant) *p* > 0.05; *p ≤ 0.05; **p ≤ 0.01. All fluorescent experiments were performed three times. The nucleus was stained by DAPI (blue); scale bar, 5 μm.

Efficient vesicular trafficking from the stage of vesicle budding to the fusion with the target membrane all depends on the dynamic regulation of the cytoskeleton. Tagln2 (transgelin2) is an actin-binding protein according to the Blast2GO Gene Ontology annotation database. When the cells were not fully stretched, Tagln2 colocalized with IFT20 around the nucleus along with accumulations in the cellular protrusions (indicated by the arrow in Figure 8A). The F-actin-like fiber localization at the cell periphery of Talgn2-mCherry was confirmed when the cells were fully stretched and co-transfected with Lifeact-EGFP, a marker of F-actin (Figure 8B). Meanwhile, Talgn2 also displayed perinuclear haze-shaped signals near the distribution of GT-EGFP (Figure 8C). Mostly, two localization patterns of Tagln2-mCherry were observed. One was a lamellipodia-localized pattern at the cell boundary, and the other was an invadopodia-localized pattern on the ventral side (Supplementary Figure 8A). Both are specialized structures used in cell migration with differences in their spatial distribution and function. Lamellipodia were generally localized at the cell front primarily for the purpose of long-distance cell migration, whereas invadopodia were localized at the cell-substratum contact points for the degradation of the extracellular matrix and promotion of cell invasion, shown as dot- or ring-shaped structures below or around the nucleus in a two-dimensional plane (Weaver, 2006). Using Strep-Tactin pulldown assays, we also detected an interaction between IFT20-strep and Tagln2-mCherry (Figure 8D). In IFT20-KO cells (B13 and A24), we detected the evident downregulation of Tagln2 (Figure 8E and Supplementary Figure 8B). Then, we generated two 4T1 cell lines stably expressing sh-Tagln2 (Supplementary Figure 8C). Tagln2-KD cells exhibited significantly faster wound closure than NC cells in the wound healing assay (Figure 8F). The number of migrating Tagln2-KD cells from the upper chamber was 3–4 times more than that of NC cells (Figure 8G). Both results implied that downregulation of Tagln2 promoted 4T1 cell migration. Taken together, we concluded that the loss of IFT20 promoted breast cancer cell migration partially by decreasing the expression of Tagln2.

## Discussion

Using non-ciliated breast cancer epithelial cells, we discovered an important cilia-independent role of IFT20, in which it functions as a negative regulator of cell migration. IFT20 not only mediates the transport of migration suppressors Numb and Ctnnal1 from the TGN to the plasma membrane, but also is associated with the dynamic regulation of F-actin through Tagln2 (Figure 8H). These results provide novel insights that can be used to understand the cell-type-specific roles of IFT20 in breast cancer cells.

The vesicle trafficking function of IFT20 was originally identified in ciliated cells based on its unique localization at the Golgi, the center of vesicle trafficking (Follit et al., 2006). GMAP210, one member of the golgins residing at the Golgi, is found to be responsible for the recruitment of cytosolic IFT20 to the cis-Golgi membrane (Follit et al., 2008). However, we did not identify this known interactor of IFT20 in our BioID results, possibly because of the varied distribution of IFT20 at the trans-Golgi/TGN in 4T1 cells, which is similar to the distribution pattern observed in photoreceptor cells (Sedmak and Wolfrum, 2010). To better understand the different sub-localizations at the Golgi with cell-cell variations, the determination of which proteins are responsible for the recruitment of IFT20 at the trans-Golgi/TGN will be an important focus of future work.

Based on the reported interplay between IFT20 and Rab-based regulatory machinery (Omori et al., 2008; Finetti et al., 2014; Su et al., 2020), we found that IFT20 participated in at least two intracellular trafficking routes—TGN-to-plasma membrane and late endosome-to-TGN—through the observable colocalization with Rab8a or Rab9. An interaction of IFT20-strep and Rab8a-mCherry was also detected in Strep-Tactin pulldown assays. It was reported that over-activated Rab8a can promote the formation of actin filaments and cell protrusions (Peranen et al., 1996; Hattula et al., 2002), similar to the phenotypes observed in our IFT20-KO cells, indicating that IFT20 and Rab8a participated in the same pathway in 4T1 cells. The co-distribution of IFT20 and Rab9 was reminiscent of the recently published results that IFT20 regulated the retrograde traffic of mannose-6-phosphate receptors (M6PRs) from the late endosomes to the TGN (Finetti et al., 2020).

Numb and Ctnnal1 are two IFT20-associated cargo proteins that were identified in the BioID method and verified through colocalization and Strep-Tactin pulldown analyses. Ctnnal1 is a scaffold protein located under the cortical actin network and functions in RhoA signaling pathway (Park et al., 2002). It has been identified in the ciliary membrane-associated proteome as an actin-binding protein (Kohli et al., 2017). Ctnnal1 on the cytoplasmic surface of IFT20-associated transport vesicles may be used to link the actin machinery, which would occur when IFT20-associated vesicles are close to the plasma membrane. Cell-type specific effect of Ctnnal1 on cell migration was reported. On one hand, Ctnnal1 could inhibit ozone-induced EMTs in bronchial epithelial cells (Tan et al., 2018). On the other hand, Ctnnal1 played a positive role in EMT and cell migration in melanoma cells (Kreiseder et al., 2013). In our study, knockdown of Ctnnal1 in 4T1 cells enhanced breast cancer cell migration, which was consistent with the effect of IFT20 depletion.

Numb is a tumor suppressor, and loss of Numb not only results in enhanced oncogenic Notch signaling but also reduced anti-oncogenic p53 expression levels (Colaluca et al., 2008). Besides the endocytic-associated function, Numb can directly bind E-cadherin. Knockdown of Numb caused a basolateral-to-apicolateral translocation of E-cadherin, a decrease in cell-cell adhesion, and an increase in cell migration in MDCK cells (Wang et al., 2009). Interestingly, despite at least four Numb mRNA splicing isoforms with different functional sites exist in mammalian cells (Dho et al., 1999; Wang et al., 2019), sequence analysis of 7 PCR-amplified products all revealed the existence of a transcript without an insert of the phosphotyrosine-binding domain in 4T1 cells, whose corresponding isoform has been reported to be not localized at the plasma membrane in MDCK cells (Dho et al., 1999). However, in our study, we observed the plasma membrane localization of this transcript, thus suggesting that some cell-type specific mechanisms (with the aid of IFT20) facilitated the transport of this Numb isoform from the TGN to the plasma membrane. This leads us to conclude that it will be necessary to identify the cell-type specific cargoes of IFT20 in different cell lines for a better understanding of its roles. Moreover, knockdown of Numb also enhanced breast cancer cell migration, which partially explained the diverse effects of IFT20 on cell migration in these cells, particularly when compared with the results in osteosarcoma and keratinocyte cells (Nishita et al., 2017; Aoki et al., 2019; Su et al., 2020).

Besides the cargoes of the IFT20-positive trafficking vesicles, we also identified a F-actin-binding protein Tagln2 in our BioID results, whose knockdown promoted 4T1 cell migration. There are two different views about the roles of Tagln2 on F-actin: depolymerizing F-actin (Leung et al., 2011) or competing with the actin-severing protein cofilin to stabilize F-actin (Na et al., 2015). In any case, Tagln2 was involved in the dynamic regulation of F-actin. Therefore, the reduced expression level of Talgn2 in IFT20-KO cells might be partially responsible for the enhanced migration.

In summary, we demonstrated an extraciliary and IFT-complex-independent role of IFT20, and established its function as a negative regulator of cell migration in breast cancer cells.

## Data Availability Statement

The original contributions presented in the study are included in the article/Supplementary Material, further inquiries can be directed to the corresponding author/s.

## Author Contributions

HY, FZ, and KH conceived and designed the study. HY, FZ, YL, and JL performed the experiments. HL provided the GT-mCherry plasmid. HY wrote the paper. KH reviewed and edited the manuscript. All authors contributed to the article and approved the submitted version.

## Conflict of Interest

The authors declare that the research was conducted in the absence of any commercial or financial relationships that could be construed as a potential conflict of interest.

## Supplementary Material

Supplementary Figure 1 Quantification of the relative expression level of IFT20 **(A)**, IFT52 **(B)**, IFT81 **(C)**, and IFT88 **(D)** normalized by the amount of β-tubulin in HBL-100, MCF-7, and MDA-MB-231 cells; all experiments were performed three times. Error bars represent the standard deviation. *P*-values indicated were calculated by using Student's *t*-tests (unpaired). n.s. *p* > 0.05; **p* ≤ 0.05; ***p* ≤ 0.01.

Supplementary Figure 2 Loss of IFT20 induces epithelial mesenchymal transitions (EMTs) in 4T1 cells. **(A)** Western blots of cell lysates from 4T1 and MEF cells probed with IFT20 antibodies. β-tubulin was used as the loading control. **(B)** Quantification of ciliated cells in 4T1 and MEF cell line under untreated condition and serum starvation for 36 h. *n* = 300 cells in three independent experiments. **(C)** The schematic diagram of the mouse *Ift20* gene with the Cas9 cleavage site indicated. **(D)** Amino acid sequence alignment of full length IFT20 in 4T1 cells and truncated IFT20 in IFT20-KO cells. **(E,F)** Quantification of the relative expression level of E-cadherin **(E)** and vimentin **(F)** normalized by the expression of β-tubulin in 4T1 and IFT20-KO cells showed that loss of IFT20 induced EMTs. Error bars represent the standard deviation. The *p*-values indicated were calculated by Student's *t*-tests (unpaired). n.s. *p* > 0.05; **p* ≤ 0.05; ***p* ≤ 0.01.

Supplementary Figure 3 Loss of IFT20 inhibits the proliferation of 4T1 cells. **(A)** Representative pictures of 4T1, B13, and A24 cells stained with crystal violet showed that the loss of IFT20 inhibited cell proliferation in the plate clone formation assay. **(B)** Quantification of the colony formation numbers in **(A)**. **(C)** Comparison of the cell proliferation in 4T1 and IFT20-KO cells by using the MTS assay showed that loss of IFT20 inhibited cell proliferation. Data are represented as the mean ± SD of three biological replicates. n.s. (not significant) *p* > 0.05; **p* ≤ 0.05; ***p* ≤ 0.01.

Supplementary Figure 4 A selective labeling strategy of GT-mCherry and GT-EGFP. **(A)** Representative fluorescent images of 4T1 cells expressing the tandem fluorescent GT-EGFP-mCherry showed that the acidic luminal environment of the trans-Golgi quenched the fluorescence of EGFP. **(B)** Representative living fluorescent images of 4T1 cells expressing GT-EGFP and stained with Golgi Tracker showed that GT-EGFP could only label part of the Golgi. **(C)** Representative fluorescent images of 4T1 cells expressing the GT-EGFP and stained with GMAP210 showed that GT-EGFP did not localize at the cis-Golgi. **(D)** Representative fluorescent images of 4T1 cells expressing the GT-mCherry and stained with GMAP210 showed that GT-mCherry did not localize at the cis-Golgi. **(E)** Representative fluorescent images of HeLa cells expressing the GT-mCherry and stained with golgin97 showed that GT-mCherry could localize at the trans-Golgi network. All fluorescent experiments were performed two **(C–E)** or three times **(A,B)**. The nucleus is stained by DAPI (blue). Scale bar, 5 μm.

Supplementary Figure 5 IFT20 participates in the anterograde transport from the TGN to the plasma membrane. **(A)** Information of Rab GTPases involved in distinct vesicle trafficking pathways. **(B)** A series of z-stack images of 4T1 cells expressing IFT20-EGFP and Rab8a-mCherry. The z-axis series of optical sections were performed at 0.8 μm-thick sections.

Supplementary Figure 6 Identification IFT20 interactors using the BioID method. **(A)** Schematic illustrating the procedures of the BioID method used to identify IFT20 interacting proteins. **(B)** Western blots of the supernatant and elute from the purification of biotinylated proteins using cell lysates from 4T1 cells expressing IFT20-BirA*-Myc or BirA*-Myc in the presence of biotin. Myc antibodies were used to show the expression of fusion proteins, and HRP-streptavidin was used to show the expression of biotinylated proteins. The supernatant included 1% of the unbound-streptavidin lysate, and the elution contained 10% of the total eluate.

Supplementary Figure 7 **(A,B)** Western blot or quantitative RT-PCR analysis showing the downregulation of Numb and Ctnnal1 corresponding to individual shRNA transfection. All experiments were performed three times. Error bars represent the standard deviation. The *p*-values indicated were calculated by using Student's *t*-tests (unpaired). n.s. (not significant) *p* > 0.05; **p* ≤ 0.05; ***p* ≤ 0.01.

Supplementary Figure 8 Tagln2-mCherry localizes at the lamellipodia and ventral invadopodia. **(A)** Representative fluorescent images of 4T1 cells expressing Tagln2-mCherry showing the lamellipodia localization or invadopodia localization of Tagln2; arrowheads indicate the lamellipodia localization at the edge of the plasma membrane; Arrows indicate the punctate invadopodia localization at the basal surface of cells. **(B)** The quantification of the relative expression level of Tagln2 normalized by the amount of β-tubulin in 4T1 and IFT20 KO cells (B13 and A24). **(C)** Quantitative RT-PCR analysis showing decreased mRNA expression levels of Tagln2 corresponding to individual shRNA transfection. All fluorescent experiments were performed three times. All quantification experiments were performed three times. Error bars represent the standard deviation. The *p*-values indicated were calculated by using Student's *t*-tests (unpaired). n.s. (not significant) *p* > 0.05; **p* ≤ 0.05; ***p* ≤ 0.01.

Supplementary Table 1 Sequence information of primers used in this study.

Supplementary Table 2 Pearson's R value between IFT20 and Rab fluorescent proteins.

Supplementary Table 3 Biotinylated protein identified in parental 4T1 cells, IFT20-DMSO and IFT20-biotin groups.

Supplementary Table 4 Biotinylated protein commonly identified in IFT20-DMSO and IFT20-biotin groups.

Supplementary Table 5 Biotinylated protein uniquely identified in IFT20-biotin group.

Supplementary Video 1 Time-lapse living video showing anterograde transport of IFT20-EGFP-associated vesicles from the cell body to the cell projection.
