# Supplementary figures and images for: IFT20 Mediates the Transport of Cell Migration Regulators From the Trans-Golgi Network to the Plasma Membrane in Breast Cancer Cells
Source: Front Cell Dev Biol. 2021 Feb 26;9:632198. doi: 10.3389/fcell.2021.632198 (PMC7968458; doi:10.3389/fcell.2021.632198)

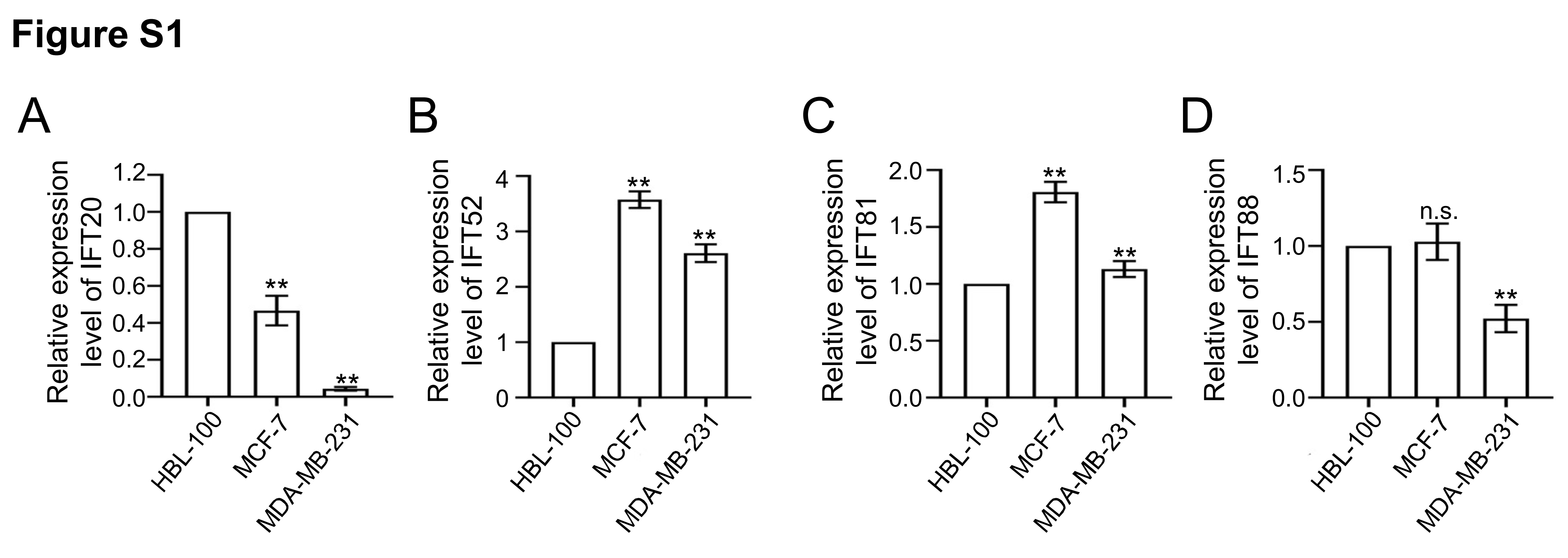

Supplement: Supplementary Figure 1 — Quantification of the relative expression level of IFT20 (A), IFT52 (B), IFT81 (C), and IFT88 (D) normalized by the amount of β-tubulin in HBL-100, MCF-7, and MDA-MB-231 cells; all experiments were performed three times. Error bars represent the standard deviation. P-values indicated were calculated by using Student's t-tests (unpaired). n.s. p > 0.05; *p ≤ 0.05; **p ≤ 0.01. [file Image_1.TIF]

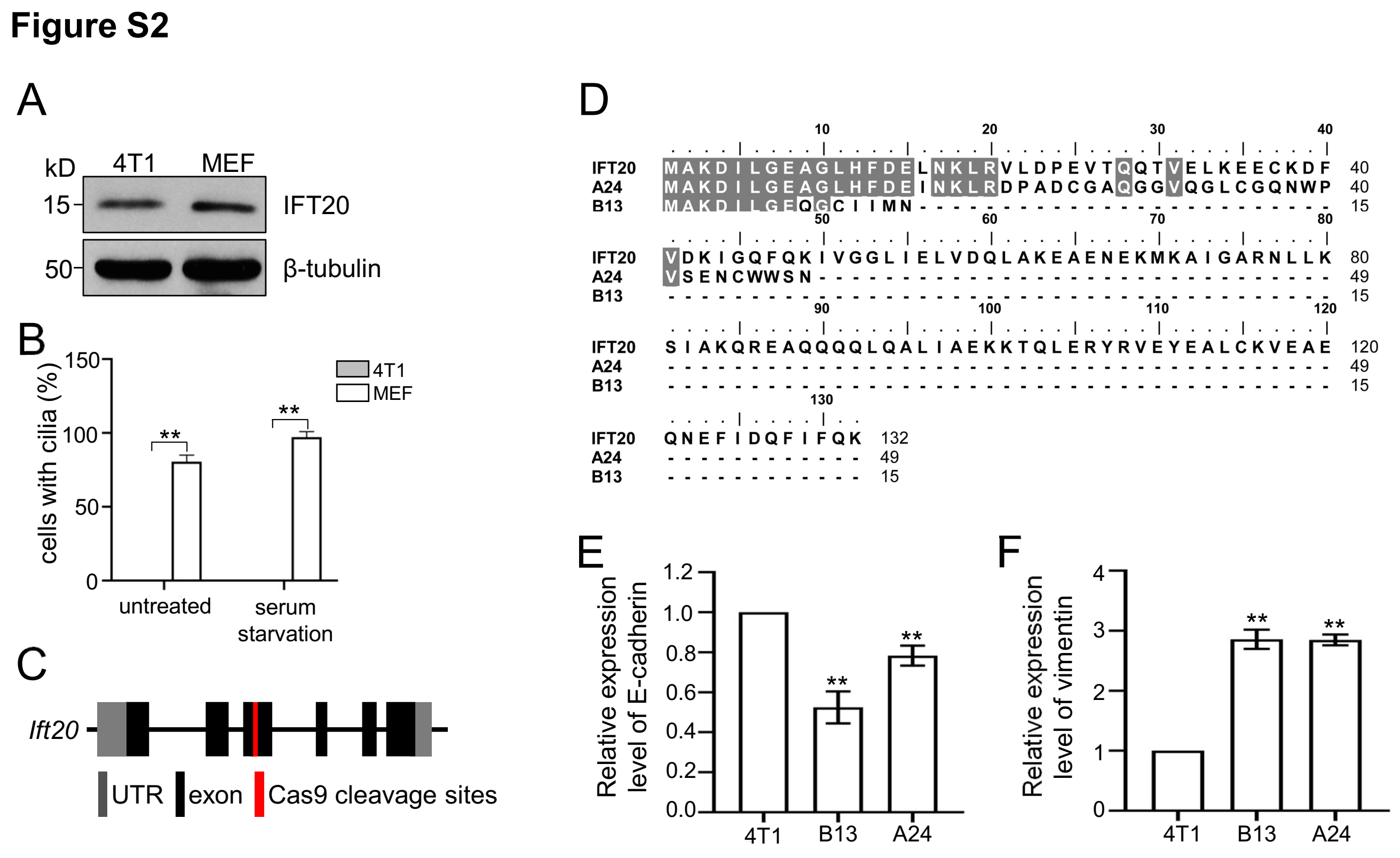

Supplement: Supplementary Figure 2 — Loss of IFT20 induces epithelial mesenchymal transitions (EMTs) in 4T1 cells. (A) Western blots of cell lysates from 4T1 and MEF cells probed with IFT20 antibodies. β-tubulin was used as the loading control. (B) Quantification of ciliated cells in 4T1 and MEF cell line under untreated condition and serum starvation for 36 h. n = 300 cells in three independent experiments. (C) The schematic diagram of the mouse Ift20 gene with the Cas9 cleavage site indicated. (D) Amino acid sequence alignment of full length IFT20 in 4T1 cells and truncated IFT20 in IFT20-KO cells. (E,F) Quantification of the relative expression level of E-cadherin (E) and vimentin (F) normalized by the expression of β-tubulin in 4T1 and IFT20-KO cells showed that loss of IFT20 induced EMTs. Error bars represent the standard deviation. The p-values indicated were calculated by Student's t-tests (unpaired). n.s. p > 0.05; *p ≤ 0.05; **p ≤ 0.01. [file Image_2.TIF]

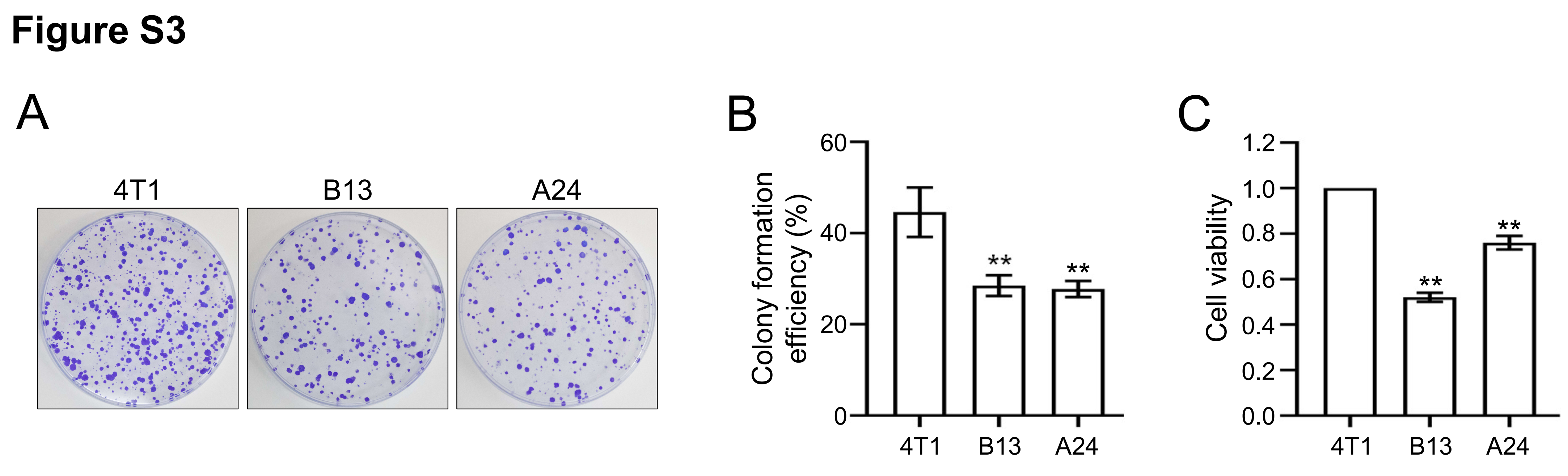

Supplement: Supplementary Figure 3 — Loss of IFT20 inhibits the proliferation of 4T1 cells. (A) Representative pictures of 4T1, B13, and A24 cells stained with crystal violet showed that the loss of IFT20 inhibited cell proliferation in the plate clone formation assay. (B) Quantification of the colony formation numbers in (A). (C) Comparison of the cell proliferation in 4T1 and IFT20-KO cells by using the MTS assay showed that loss of IFT20 inhibited cell proliferation. Data are represented as the mean ± SD of three biological replicates. n.s. (not significant) p > 0.05; *p ≤ 0.05; **p ≤ 0.01. [file Image_3.TIF]

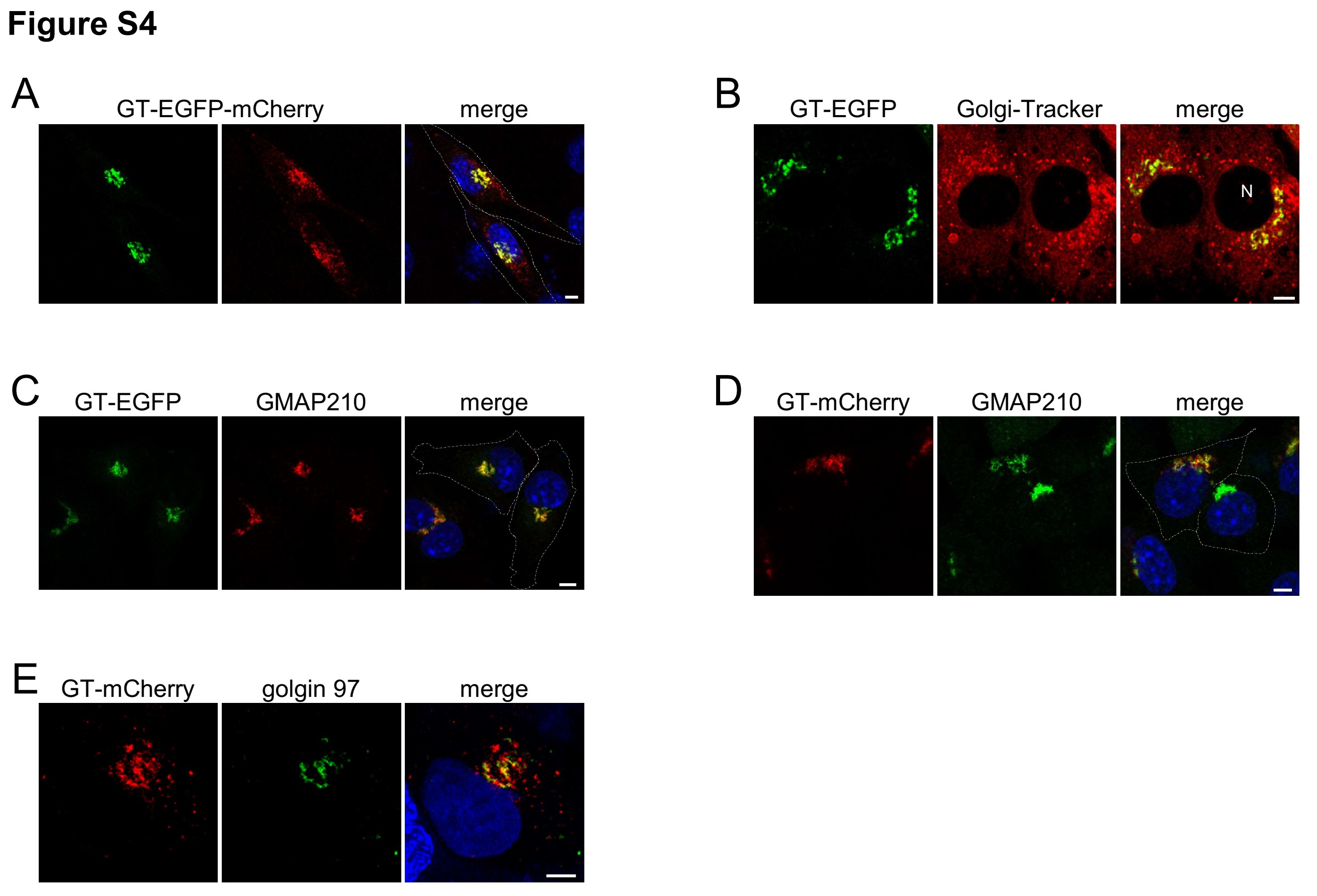

Supplement: Supplementary Figure 4 — A selective labeling strategy of GT-mCherry and GT-EGFP. (A) Representative fluorescent images of 4T1 cells expressing the tandem fluorescent GT-EGFP-mCherry showed that the acidic luminal environment of the trans-Golgi quenched the fluorescence of EGFP. (B) Representative living fluorescent images of 4T1 cells expressing GT-EGFP and stained with Golgi Tracker showed that GT-EGFP could only label part of the Golgi. (C) Representative fluorescent images of 4T1 cells expressing the GT-EGFP and stained with GMAP210 showed that GT-EGFP did not localize at the cis-Golgi. (D) Representative fluorescent images of 4T1 cells expressing the GT-mCherry and stained with GMAP210 showed that GT-mCherry did not localize at the cis-Golgi. (E) Representative fluorescent images of HeLa cells expressing the GT-mCherry and stained with golgin97 showed that GT-mCherry could localize at the trans-Golgi network. All fluorescent experiments were performed two (C–E) or three times (A,B). The nucleus is stained by DAPI (blue). Scale bar, 5 μm. [file Image_4.TIF]

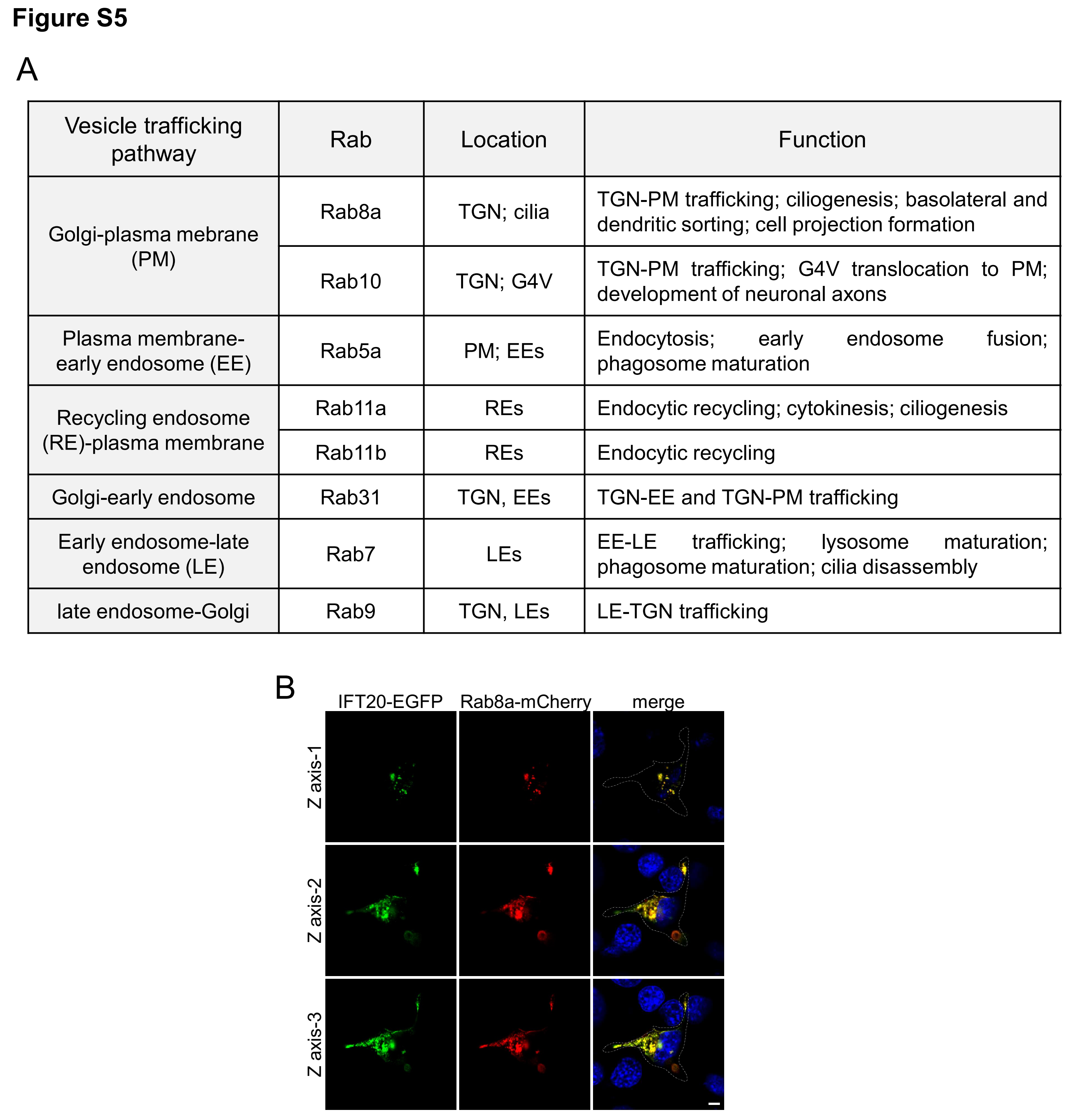

Supplement: Supplementary Figure 5 — IFT20 participates in the anterograde transport from the TGN to the plasma membrane. (A) Information of Rab GTPases involved in distinct vesicle trafficking pathways. (B) A series of z-stack images of 4T1 cells expressing IFT20-EGFP and Rab8a-mCherry. The z-axis series of optical sections were performed at 0.8 μm-thick sections. [file Image_5.TIF]

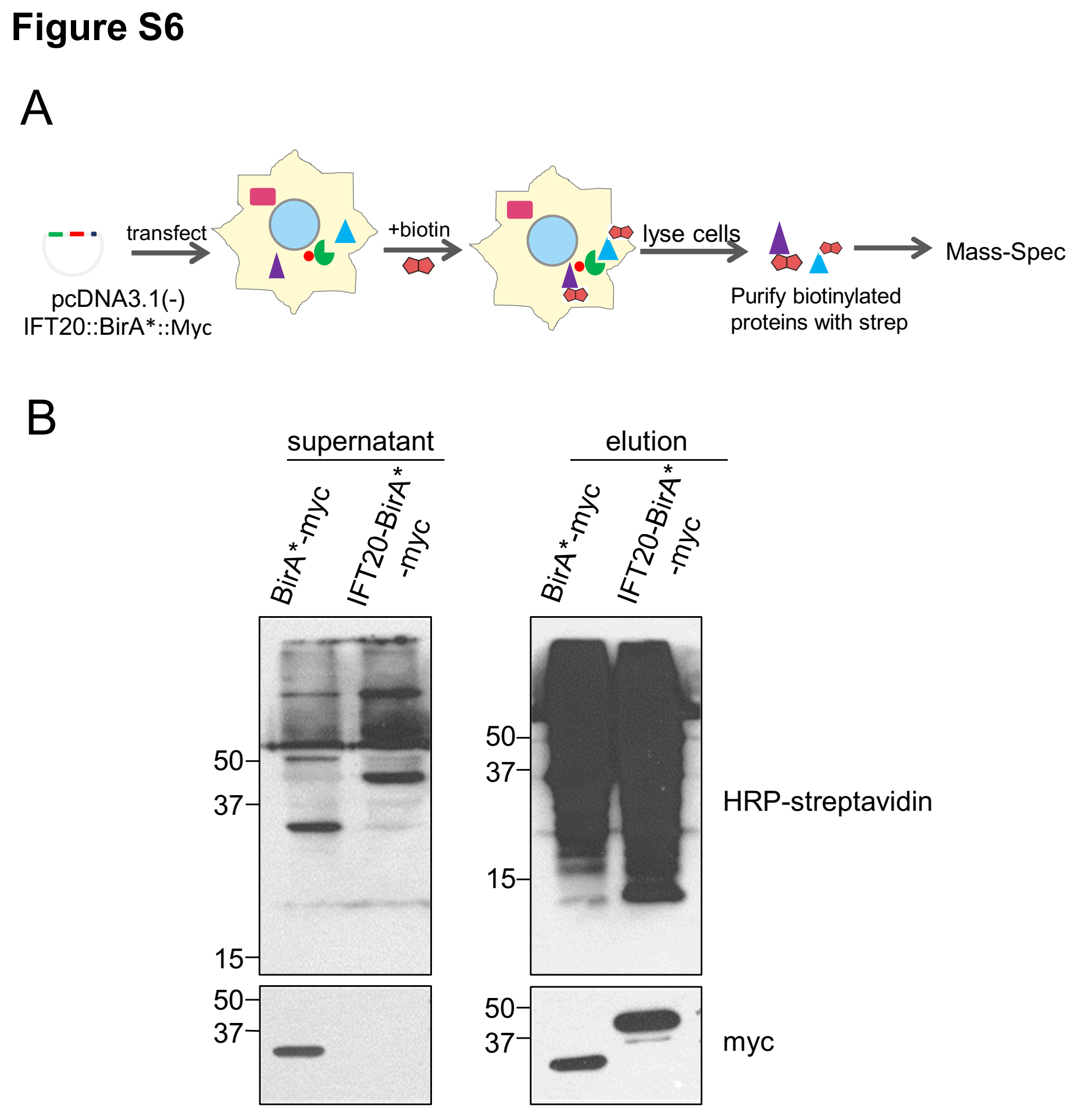

Supplement: Supplementary Figure 6 — Identification IFT20 interactors using the BioID method. (A) Schematic illustrating the procedures of the BioID method used to identify IFT20 interacting proteins. (B) Western blots of the supernatant and elute from the purification of biotinylated proteins using cell lysates from 4T1 cells expressing IFT20-BirA*-Myc or BirA*-Myc in the presence of biotin. Myc antibodies were used to show the expression of fusion proteins, and HRP-streptavidin was used to show the expression of biotinylated proteins. The supernatant included 1% of the unbound-streptavidin lysate, and the elution contained 10% of the total eluate. [file Image_6.TIF]

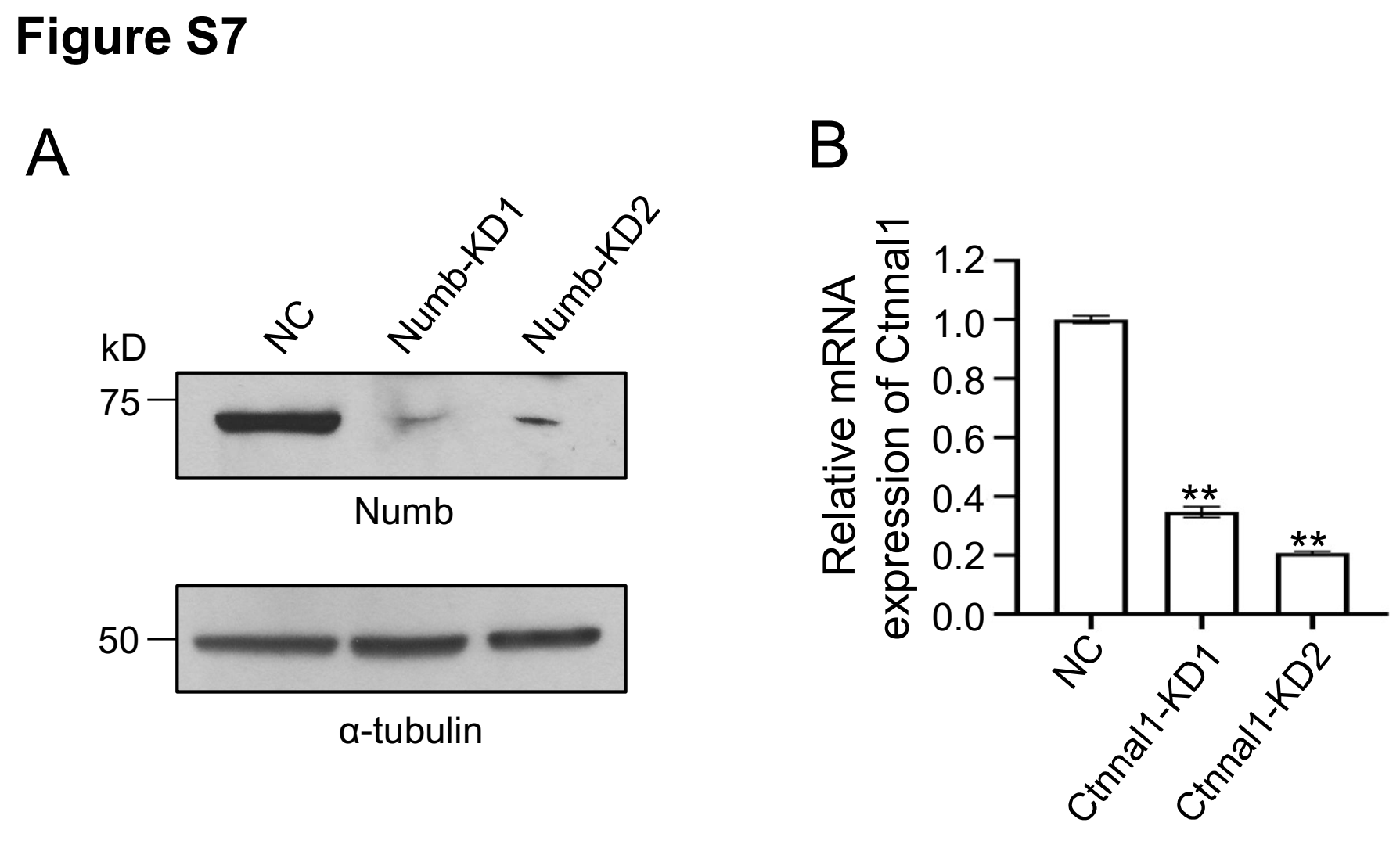

Supplement: Supplementary Figure 7 — (A,B) Western blot or quantitative RT-PCR analysis showing the downregulation of Numb and Ctnnal1 corresponding to individual shRNA transfection. All experiments were performed three times. Error bars represent the standard deviation. The p-values indicated were calculated by using Student's t-tests (unpaired). n.s. (not significant) p > 0.05; *p ≤ 0.05; **p ≤ 0.01. [file Image_7.TIF]

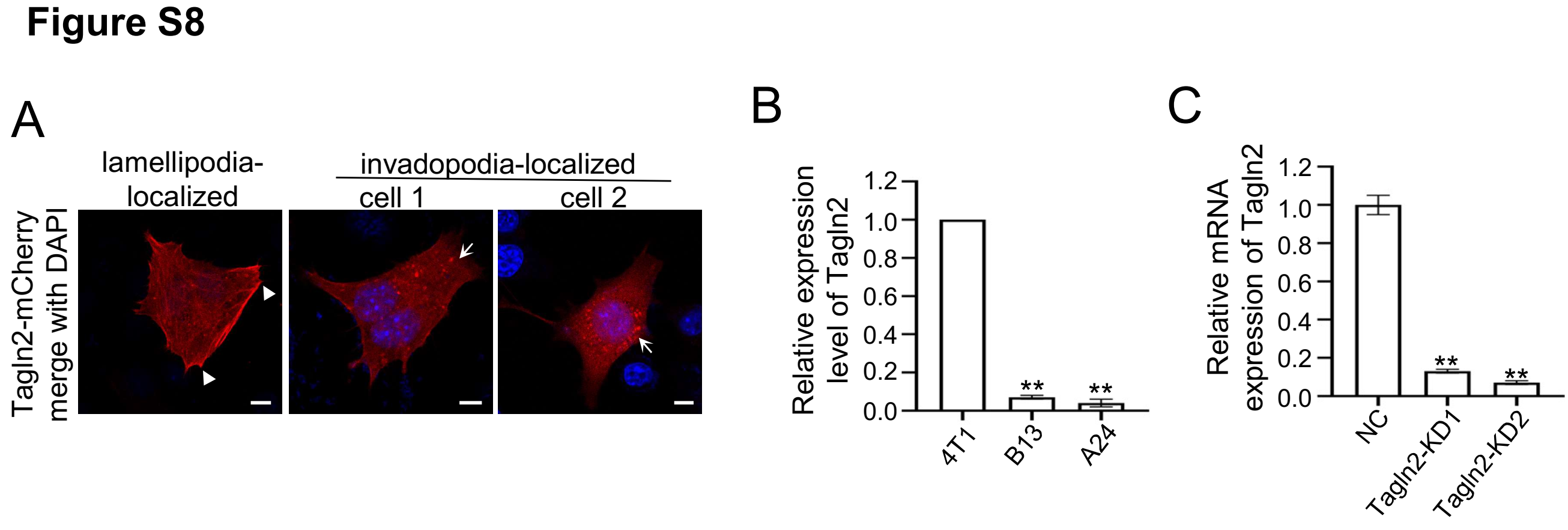

Supplement: Supplementary Figure 8 — Tagln2-mCherry localizes at the lamellipodia and ventral invadopodia. (A) Representative fluorescent images of 4T1 cells expressing Tagln2-mCherry showing the lamellipodia localization or invadopodia localization of Tagln2; arrowheads indicate the lamellipodia localization at the edge of the plasma membrane; Arrows indicate the punctate invadopodia localization at the basal surface of cells. (B) The quantification of the relative expression level of Tagln2 normalized by the amount of β-tubulin in 4T1 and IFT20 KO cells (B13 and A24). (C) Quantitative RT-PCR analysis showing decreased mRNA expression levels of Tagln2 corresponding to individual shRNA transfection. All fluorescent experiments were performed three times. All quantification experiments were performed three times. Error bars represent the standard deviation. The p-values indicated were calculated by using Student's t-tests (unpaired). n.s. (not significant) p > 0.05; *p ≤ 0.05; **p ≤ 0.01. [file Image_8.TIF]
